# Combined homologous recombination repair deficiency and immune activation analysis for predicting intensified responses of anthracycline, cyclophosphamide and taxane chemotherapy in triple-negative breast cancer

**DOI:** 10.1186/s12916-021-02068-4

**Published:** 2021-09-01

**Authors:** Gaoming Liao, Zedong Jiang, Yiran Yang, Cong Zhang, Meiting Jiang, Jiali Zhu, Liwen Xu, Aimin Xie, Min Yan, Yunpeng Zhang, Yun Xiao, Xia Li

**Affiliations:** 1 College of Bioinformatics Science and Technology, Harbin Medical University, Harbin, 150081 Heilongjiang China; 2 Department of Ultrasonic Medicine, The First Affiliated Hospital of Harbin Medical University, Harbin, 150010 Heilongjiang China; 3 Key Laboratory of University in Heilongjiang Province, Department of Pharmacy, The Second Affiliated Hospital of Harbin Medical University, Harbin, 150086 China; 4 Key Laboratory of Cardiovascular Medicine Research, Harbin Medical University, Ministry of Education, Harbin, 150081 Heilongjiang China

**Keywords:** Triple-negative breast cancer, ACT chemotherapy, Homologous recombination repair deficiency, Failure-free interval, Immune checkpoint

## Abstract

**Background:**

Triple-negative breast cancer (TNBC) is a clinically aggressive disease with abundant variants that cause homologous recombination repair deficiency (HRD). Whether TNBC patients with HRD are sensitive to anthracycline, cyclophosphamide and taxane (ACT), and whether the combination of HRD and tumour immunity can improve the recognition of ACT responders are still unknown.

**Methods:**

Data from 83 TNBC patients in The Cancer Genome Atlas (TCGA) was used as a discovery cohort to analyse the association between HRD and ACT chemotherapy benefits. The combined effects of HRD and immune activation on ACT chemotherapy were explored at both the genome and the transcriptome levels. Independent cohorts from the Molecular Taxonomy of Breast Cancer International Consortium (METABRIC) and Gene Expression Omnibus (GEO) were adopted to validate our findings.

**Results:**

HRD was associated with a longer ACT chemotherapy failure-free interval (FFI) with a hazard ratio of 0.16 (*P* = 0.004) and improved patient prognosis (*P* = 0.0063). By analysing both HRD status and ACT response, we identified patients with a distinct TNBC subtype (ACT-S&HR-P) that showed higher tumour lymphocyte infiltration, IFN-γ activity and NK cell levels. Patients with ACT-S&HR-P had significantly elevated immune inhibitor levels and presented immune activation associated with the increased activities of both innate immune cells and adaptive immune cells, which suggested treatment with immune checkpoint blockade as an option for this subtype. Our analysis revealed that the combination of HRD and immune activation enhanced the efficiency of identifying responders to ACT chemotherapy (AUC = 0.91, *P* = 1.06e−04) and synergistically contributed to the clinical benefits of TNBC patients. A transcriptional HRD signature of ACT response-related prognostic factors was identified and independently validated to be significantly associated with improved survival in the GEO cohort (*P* = 0.0038) and the METABRIC dataset (*P* < 0.0001).

**Conclusions:**

These findings highlight that HR deficiency prolongs FFI and predicts intensified responses in TNBC patients by combining HRD and immune activation, which provides a molecular basis for identifying ACT responders.

**Supplementary Information:**

The online version contains supplementary material available at 10.1186/s12916-021-02068-4.

## Background

Triple-negative breast cancer (TNBC) characterized by absent or minimal expression of oestrogen receptors (ER), progesterone receptors (PR) and human epidermal growth factor receptor 2 (HER2) is a highly heterogeneous and aggressive disease and has the worst prognosis among the different subtypes of breast cancer [1, 2]. Among the chemotherapy regimens used for TNBC, sequential anthracycline (A) and cyclophosphamide (C) followed by taxane (T) (ACT) are some of the preferred regimens in international guidelines [3, 4]. However, approximately 30–40% of patients with residual disease (RD) after surgery treated with ACT-based therapy will develop metastatic disease and death [5].

The detailed molecular characterization of refractory tumours is a prerequisite to understanding therapy resistance and developing reasonable treatment strategies. Homologous recombination repair (HRR) is a high-fidelity repair mechanism specifically for DNA double-strand breaks (DSBs) [6]. *BRCA1/2* are key components in the HR-mediated DNA DSB repair mechanism, and mutations in *BRCA1/2* are typical molecular alterations that lead to homologous recombination repair deficiency (HRD) and sensitivity to DNA damage agents [7, 8]. In vitro and preclinical studies have shown that tumours with HR deficiency are sensitive to platinum-containing and/or DNA damage mutagens, which significantly increases the patient response rate and prolongs survival [9, 10]. However, whether HR deficiency could improve the response to DNA-damaging or repair-inhibiting therapies such as doxorubicin (which induces DNA DSBs) and cyclophosphamide (an alkylating agent that causes DNA crosslinks that lead to DSBs) [8, 10] in TNBCs remains poorly characterized, although *BRCA1/2* germline mutations have been shown to promote the pathological complete response (pCR) in early TNBC patients who received ACT chemotherapy [11].

Among chemotherapy options in TNBC, combining immunomodulatory therapy (such as atezolizumab) with nab-paclitaxel and anthracycline-based chemotherapy is potentially advantageous, significantly improving pCR rates with an acceptable safety profile [12, 13]. Shibata et al. showed that the DNA DSB repair pathway upregulated the *PD-L1* expression in cancer cells by activating *STAT1* and *STAT3* signalling and the *IRF1* pathway [14]. Unrepaired DSBs regulate the tumour immune microenvironment through a series of molecular and cellular mechanisms, such as increasing genomic instability, activating immune pathway activation and facilitating *PD-L1* expression on cancer cells, which might promote responsiveness to immune checkpoint inhibitors (ICIs) [15, 16]. The clinical and translational data indicated that low-dose chemotherapy may be utilized to stimulate anticancer immune responses. For example, short-term doxorubicin treatment may promote a more favourable tumour microenvironment and increase the likelihood of a response to *PD-1* blockade in TNBC [17]. Additionally, low-dose cyclophosphamide induced antitumour T cell responses in metastatic colorectal cancer [18]. These studies indicated that it is necessary to consider the mechanism of the tumour immune microenvironment to characterize the effect of HRD on the ACT chemotherapy response.

Here, we performed an integrated genome analysis of TNBC patients who received ACT treatment after tumour resection, specifically focusing on HRD and the immune microenvironment and their combined effect on the treatment response and clinical outcomes. A transcriptional HRD signature as a prognostic factor related to ACT chemotherapy was identified and independently validated to be significantly associated with improved survival in the GEO cohorts and the METABRIC dataset. Our goal is to evaluate the combination of HRD and immune activation as a potentially stronger tool to predict which TNBC patients might achieve a valuable response to ACT-based preoperative chemotherapy.

## Methods

### Sample collection and datasets

#### Discovery cohort

**Fig. 1:**
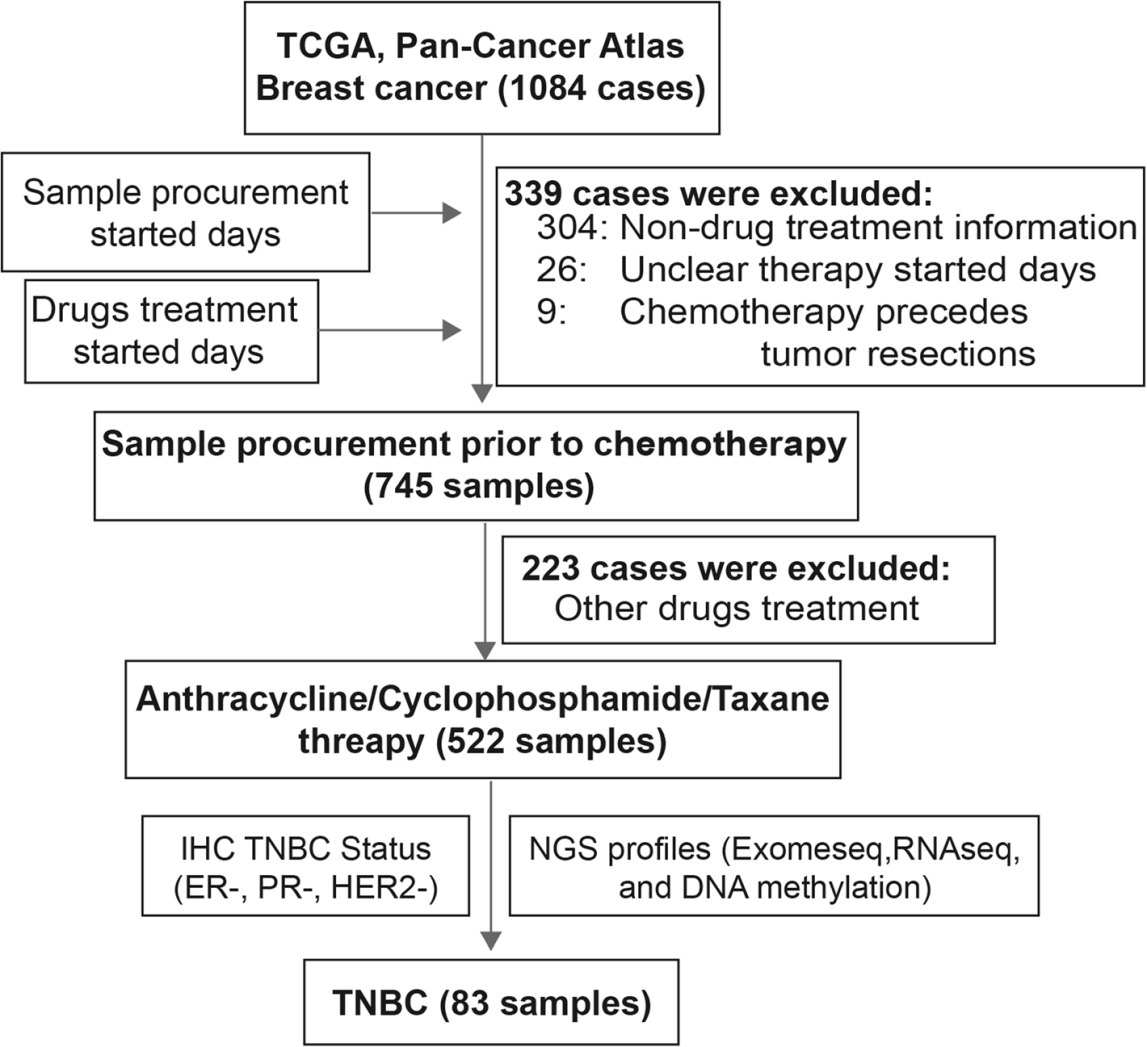
Sampling procedure for TNBC patients prior to ACT chemotherapy

In this study, we downloaded whole-exome sequencing (WES) data and gene expression profiles (RNA-Seq, RSEM standardization) for breast cancer patients in The Cancer Genome Atlas (TCGA) from cBioPortal [19]. Additionally, the patient survival and clinical phenotype (including age, PAM50 subtype and AJCC tumour stage) data were obtained. The sampling time points of patients and medication information related to the anticancer drugs used (including start/end time points of treatment) were obtained from the Genomic Data Commons Data Portal (GDC Data Portal). We reviewed the drug names and manually standardized them through DrugBank [20]. According to the dates given for sampling and first treatment, we obtained a clear cohort of primary breast cancer patients who received ACT treatments after tumour resection (Fig. 1). After determining the immunohistochemistry (IHC) status of ER, PR and HER2 and selecting patients for whom exome sequencing data were available, we established a final cohort of 83 TNBC patients (Table 1). Patients who were sensitive to ACT treatment were defined as having complete responses to ACT or a failure-free interval (FFI) above the median. Patients with disease progression or no improvement after ACT treatment ended (progressive disease/stable disease), or an FFI below the median were defined as resistant to ACT (Table 1).

**Table 1:** Patient and tumour characteristics of TNBC

Baseline characteristics (***n*** = 83)	***N*** (% or range)
**Age (mean)**	52 (29–78)
**AJCC stage**	
Stage I, stage IA	18 (21.7)
Stage IIA, stage IIB	52 (62.7)
Stage IIIA, stage IIIB, stage IIIC	12 (14.5)
**PAM50 subtype**	
Basal	64 (80.7)
Her2	5 (6.0)
LumB	1 (1.2)
**HRD status**	
HR deficiency	48 (57.8)
HR proficiency	35 (42.2)
***BRCA1/2*mutation**	
*BRCA1*	6 (7.2)
*BRCA2*	5 (6.0)
***BRCA1*promoter methylation**	7 (7.2)
**ACT chemotherapy response**	
Complete response	21 (25.3)
Partial response	2 (2.4)
Progressive disease	1 (1.2)
–	59 (71.1)
**ACT response status**	
Sensitive	39 (47.0)
Resistant	44 (53.0)

#### Validation cohorts

We downloaded data from GEO (GSE25055, GSE25065 and GSE41998), including expression profile, ACT response and clinical phenotype, for validation cohorts of TNBC patients who received neoadjuvant ACT therapy after sample procurement [21, 22]. A cohort of 299 TNBC patients who received ACT chemotherapy after tissue sample collection was obtained from METABRIC [23]. In addition, two cohorts of TNBC patients (Hess2006, neoadjuvant therapy; Chin2006, adjuvant therapies) who were treated with ACT after sample procurement were acquired from UCSC Xena [24, 25]. Hess2006 cohort patients undergoing diagnostic biopsy were prior to ACT neoadjuvant therapy. Information on all TNBC patients used in this study is provided in Additional file 1: Table S1.

### HRD score, mutational signature and HRDetect score

The HRD score was calculated based on the number of specific lesions in the genome, including loss-of-heterozygosity (LOH), large-scale transitions (LST) and the number of telomeric allelic imbalances (ntAI). Following Thorsson et al.’s research on TCGA data [26], we extracted the HRD scores of TNBC patients. Somatic point mutational signatures were determined with the deconstructSigs R package [27] by using the COSMIC signatures as a mutational-process matrix. The WES-based HRDetect scores were calculated using the lasso logistic model by fitting multiple predictors related to HRD, including the HRD score, the contribution of major mutational signatures in breast cancers (such as signature 1, signature 3, signature 6 and signature 20) and the insertion/deletion ratio [28]. The weights of the whole exome–specific model were trained on 560 artificial whole exomes [29].

### *BRCA1* promoter methylation analysis

We acquired DNA methylation data (Illumina Human Methylation 450) from the UCSC Xena database. The transcription start site (TSS) information of the human reference genome (GRCh38) was obtained from Ensembl. The promoter region was defined as the 1500 bp upstream and 500 bp downstream of the TSS. We identified a robust probe (cg13782816) for the *BRCA1* gene located in the promoter region through the gene symbol annotation information. *BRCA1* promoter methylation (epigenetic silencing) was defined as a cg13782816 methylation level exceeding 0.9.

### Immune microenvironment mechanism of TNBC patients

The R package CIBERSORT [30] was used to calculate the level of immune cell infiltration in TNBC patients. The IFN-γ score of the TNBC patients was calculated using the Tumor Immune Dysfunction and Exclusion (TIDE) online analysis tool [31]. The immune molecular and cellular characteristics of TCGA BRCA patients were obtained from a previous study [26], including tumour lymphocyte infiltration (TLI) score, regulatory macrophages (Mregs), TCR/BCR Shannon, TCR/BCR richness and TGF-β response and neoantigens. Tumour mutation burden (TMB) was defined as the total number of nonsynonymous single nucleotide and indel variants.

### Gene set enrichment analysis and pathway activity calculation

We downloaded all the pathways of Collection 2 (C2) and their included gene sets from MSigDB (v7.2) [32]. Patients with the ACT-S&HR-P subtype served as the case group, other patients served as the control group and the R package DESeq2 was used for differential expression analysis. Gene set enrichment analysis (GSEA) was performed using the R package clusterProfiler. We acquired the gene sets of both immune cell types and core biological pathways from previous studies [33, 34] and calculated the activity of these pathways/immune cells utilizing the R package GSVA. We obtained sets of immunostimulators and immune inhibitors based on known studies [35, 36] and explored the differences in the expression of these genes between patients with ACT-S&HR-P and other TNBCs.

### Immune score of TNBC patients

Two different methods were used to calculate the immune score (IS) of each TNBC patient based on the gene expression levels. (1) Due to the significantly higher activities of immune response-related pathways (including interferon and immune checkpoint blockade-related pathways, as well as CD8 effector T cells) in the ACT-S&HR-P subtype, we calculated the IS according to the sum of the activities of these pathways (Additional file 1: Table S2). (2). The IS was also calculated using the average expression of prognostic immune markers in breast cancer [37].

### Identification of the HRD expression signature and computation of the HRD-related prognostic score

After grouping TNBC samples (HR deficiency and HR proficiency) according to HRD status and performing differential expression analysis using the R package DESeq2, we identified differentially expressed genes (DEGs) using FDR ≤ 0.05 and fold change ≥ 2 or ≤ 1/2 as the threshold. Univariate Cox regression analysis was performed in the FFI of TNBC patients based on the expression levels of all DEGs. Finally, 15 HRD-related prognostic factors (HRD expression signature) were determined after removing redundant factors using lasso logistic regression. Among those signature genes, 4 were strongly overexpressed (FDR ≤ 0.05, fold change ≥ 2), and 11 were downregulated (FDR ≤ 0.05, fold change ≤ 1/2) in HR-deficient compared to HR-proficient cases.

Considering the transcription levels of the HRD expression signature and the hazard ratio calculated by Cox regression analysis, the prognostic score (PS) of upregulated factors (all hazard ratio < 1, protective factor) and downregulated factors (all hazard ratio > 1, risk factor) were computed as follows:

$$ {\mathrm{PS}}_i\kern0.5em =\kern0.5em \sum \limits_{j=1}^n\frac{{\mathrm{Exp}}_{ij}}{{\mathrm{HR}}_j} $$

where HR_*j*_ represents the hazard ratio of upregulated (or downregulated) factor *j* in the Cox model. Exp_*ij*_ represents the expression levels (log-transformed) of upregulated (or downregulated) factor *j* in sample *i*. We calculated the HRD-related prognostic score (HRDPS) of patients based on the PS of both upregulated and downregulated factors as follows:

$$ {\mathrm{HRDPS}}_i\kern0.5em =\kern0.5em {\mathrm{PS}}_{i,\mathrm{up}}\kern0.5em -\kern0.5em {\mathrm{PS}}_{i,\mathrm{down}} $$

where PS_*i*, up_ represents the prognostic score of upregulated factors in sample *i*, and PS_*i*, down_ represents the prognostic score of downregulated factors in sample *i*.

### Statistical analyses

R Project (version 4.02) for statistical computing was used in this study. The nonparametric Wilcoxon rank-sum test was used to explore the statistical significance between discrete variables (such as HRD status and ACT response status) and continuous indicators (such as immune cell infiltration and pathway activities). The nonparametric Kruskal-Wallis test was used for comparisons among multiple groups, with Benjamini and Hochberg false discovery rate (FDR) correction. For some comparisons (activated NK cells, M0 macrophages and activated mast cells), we additionally performed a combinatorial method that Wilcoxon’s rank-sum test with continuity correction combined 10,000 iterations. Fisher’s exact test was used to examine the relationships between HRD status and HRR-related gene mutations, *BRCA1* promoter methylation and the effect on the response to ACT chemotherapy. Clinical outcomes and FFI were compared using the log-rank test. Kaplan-Meier graphs were plotted using standard methodologies. A Cox proportional hazards model was used to calculate the hazard ratios and corresponding 95% confidence intervals (CIs) with adjustments for age, tumour stage and disease stage. Statistical significance was set at two-tailed *P* < 0.05.

## Results

### Genomic alterations and homologous recombination repair defects were widespread in TNBC patients

**Fig. 2:**
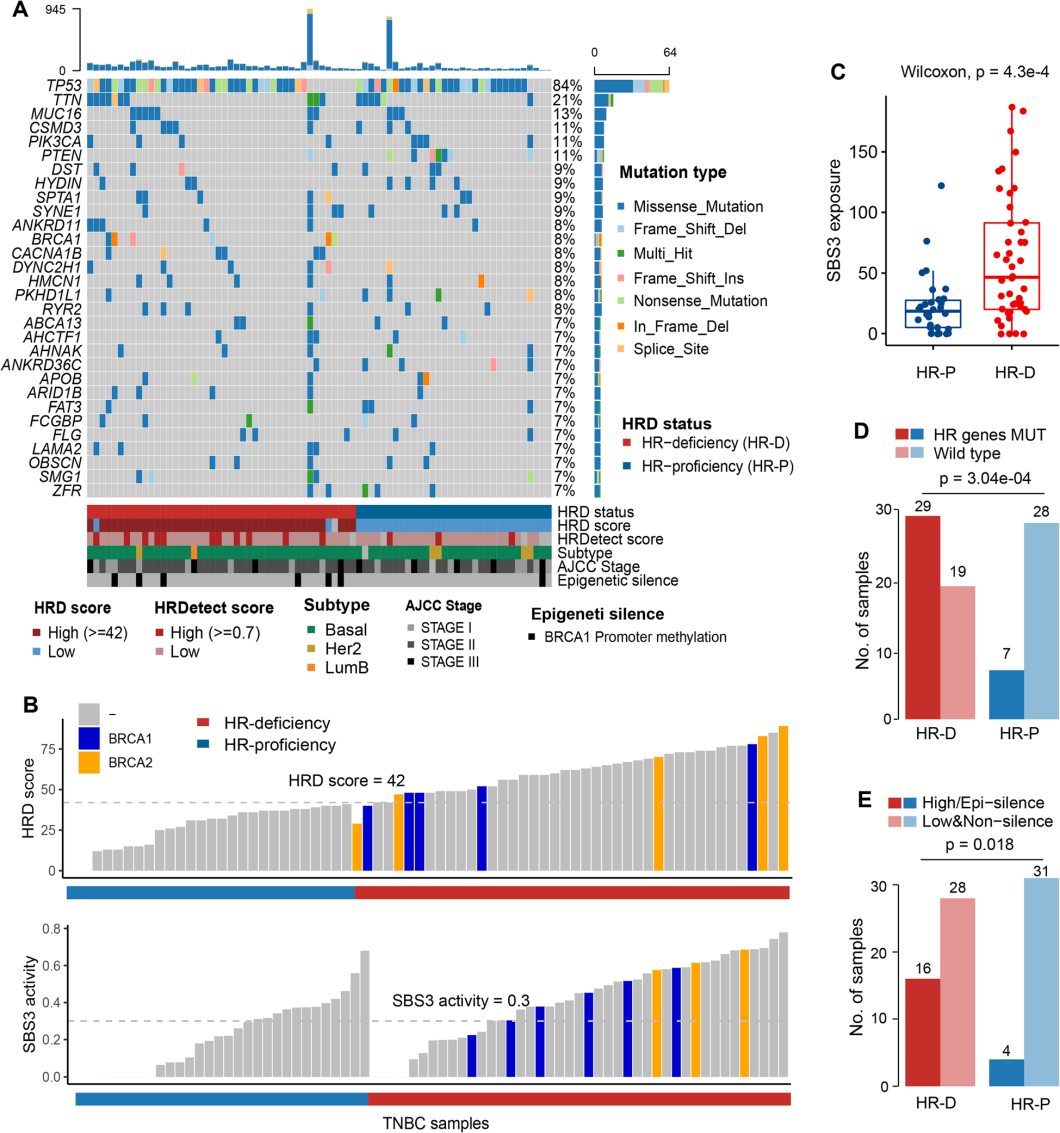
Genome alterations and HRDs in TNBC patients. **A** Mutation profile of high-frequency mutation genes in patients (top 30). **B** Distribution of HRD score and mutational signature 3 activity in patients with HR deficiency and HR proficiency. The patients with *BRCA1/BRCA2* mutations highlighted in blue and orange colours, respectively. **C** Box plot showing the distribution of mutational signature 3 exposure in patients with HR deficiency (HR-D) and HR proficiency (HR-P). SBS3, single-base substitution signature 3. *P*-value was calculated by the Wilcoxon rank-sum test. **D**, **E** The bar graph showing the relationship between HRD status and HRR gene mutation (**D**), *BRCA1* promoter methylation/HRDetect score and HRDetect score^hi^ (≥ 0.7) (**E**). *P*-value was calculated by Fisher’s exact test

We acquired data for 83 TNBC patients who underwent ACT chemotherapy after tissue sample collection based on the dates given for sampling and first treatment (Fig. 1). Consistent with a previous study [38], most TNBC patients belonged to the basal subtype (88%; Fig. 2A). As expected, TNBC patients carrying mutations in *TP53* (84%), *PTEN* (11%) and *BRCA1/2* (8% and 7%, respectively) were relatively frequent among breast cancer (BC) patients in the PanCancer Atlas [39] (Fig. 2A, Additional file 1: Fig. S1A). In contrast, the oncogene *PIK3CA* (11%) was less prone to the mutation in TNBCs than all BC patients.

Biomarkers for homologous recombination repair deficiency in cancers have attracted great interest from researchers [8, 40]. HR deficiency was defined as either a deleterious tumour *BRCA1/2* (tBRCA) mutation or a predefined HRD score ≥ 42 [8], which was determined in 57.8% (48/83) of TNBC patients (Fig. 2A, Table 1). Our results showed that most TNBC patients with *BRCA1/2* mutations presented HRD scores^high^ (≥ 42) and dominant mutational signature 3 (SBS3) activity (≥ 0.3) (both 8/11; Fig. 2B). Indeed, the patients with HR deficiency showed significantly higher SBS3 exposure than patients with HR proficiency (*P* = 4.3e−04, Wilcoxon rank-sum test; Fig. 2C). In addition to *BRCA1/2*, several important components of the HRR pathway, such as *RAD54* family members (including *RAD54B* and *RAD54L*), DNA polymerase members (including *POLD1*, *POLH* and *POLQ*) and *PARP1*, *PALB2* and *TP53BP1*, occurred mainly in the HR deficient samples (29/48, *P* = 3.04e−04, Fisher’s exact test; Fig. 2D, Additional file 1: Fig. S1B). Consistent with previous studies, we also found that HR-deficient patients showed a higher proportion of *BRCA1* promoter methylation and HRDetect score^high^ (16/48, *P* = 0.018, Fisher’s exact test; Figs. 2A, E) [28, 41]. These results indicated that HRD could be characterized by mutations in HRR-related genes, SBS3 exposure and *BRCA1* promoter hypermethylation.

### Homologous recombination repair deficiency correlates with ACT chemotherapy benefits

**Fig. 3:**
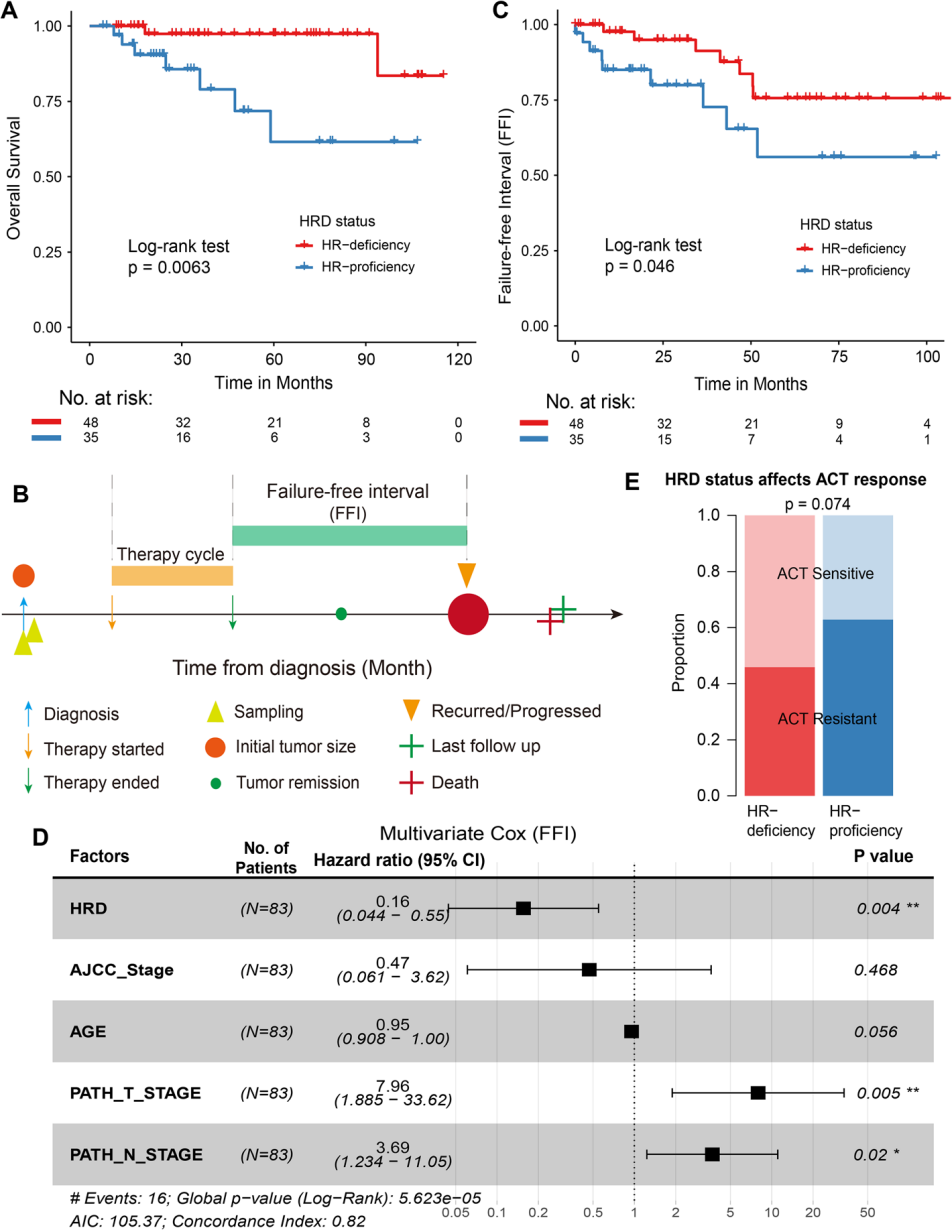
Homologous recombination repair deficiency correlates with ACT chemotherapy benefits. **A**, **C** Kaplan-Meier graphs of HRD status on OS (**A**) and FFI (**C**). Statistical significance was calculated using the log-rank test. **B** Conceptual depiction of the TNBC patients undergoing ACT chemotherapy. **D** Forest plot illustrating the HR (95% CI) for FFI calculated using the multivariate Cox proportional hazard models. HR, hazard ratios; CI, confidence interval. **E** Distribution of ACT sensitive (light colour) or resistant (dark colour) in patients with HR deficiency (red) and HR proficiency (blue)

Accumulated evidence has shown that HRD is associated with a better prognosis for patients with a variety of solid tumours [42, 43]. Whether a better prognosis is related to HRD in TNBC patients who received ACT chemotherapy has not been well characterized. Our results revealed that compared with patients with HR proficiency, patients with HR deficiency showed significantly better overall survival (OS; *P* = 0.0063, log-rank test; Fig. 3A) and disease-specific survival (DSS; *P* = 0.023, log-rank test; Additional file 1: Fig. S2A) after treatment with ACT. Specifically, the 5-year OS rate for HR-deficient patients was 98%, while that for HR-proficient patients, it was only 61% (Fig. 3A). Multivariate Cox regression analysis showed that HR deficiency was an independent protective factor associated with prolonged patient OS (*P* = 0.002, log-rank test; Additional file 1: Fig. S2B) and DSS (*P* = 0.014, log-rank test; Additional file 1: Fig. S2C) in TNBC after adjusting for clinical factors including age, AJCC stage and TNM stage.

Furthermore, to explore whether HR deficiency can indeed benefit TNBC patients’ response to ACT treatment, we determined the ACT chemotherapy failure-free interval (FFI) of patients based on the period from the end of treatment to tumour progression/recurrence or death (Fig. 3B). Our results showed that HR deficiency was correlated with durable response to ACT chemotherapy (*P* = 0.046, log-rank test; Fig. 3C). The 5-year FFI rate for HR-deficient patients was 75%, while that for HR proficiency, it was only 52%. Cox regression analysis showed that HR deficiency was a significant protective factor for FFI in TNBC patients with a hazard ratio of 0.16 (95% CI 0.044–0.55, *P* = 0.004; Fig. 3D), which improved the response interval to ACT chemotherapy. In addition, we found that HR-deficient patients tended to be more sensitive to ACT chemotherapy (54.2% for sensitive, 37.1% for resistant), compared with HR-proficient patients (37.1% for sensitive, 62.9% for resistant; *P* = 0.074, Fisher’s exact test; Fig. 3E).

### Revealing the diversity of the immune microenvironment related to ACT responses utilizing HRD status

**Fig. 4:**
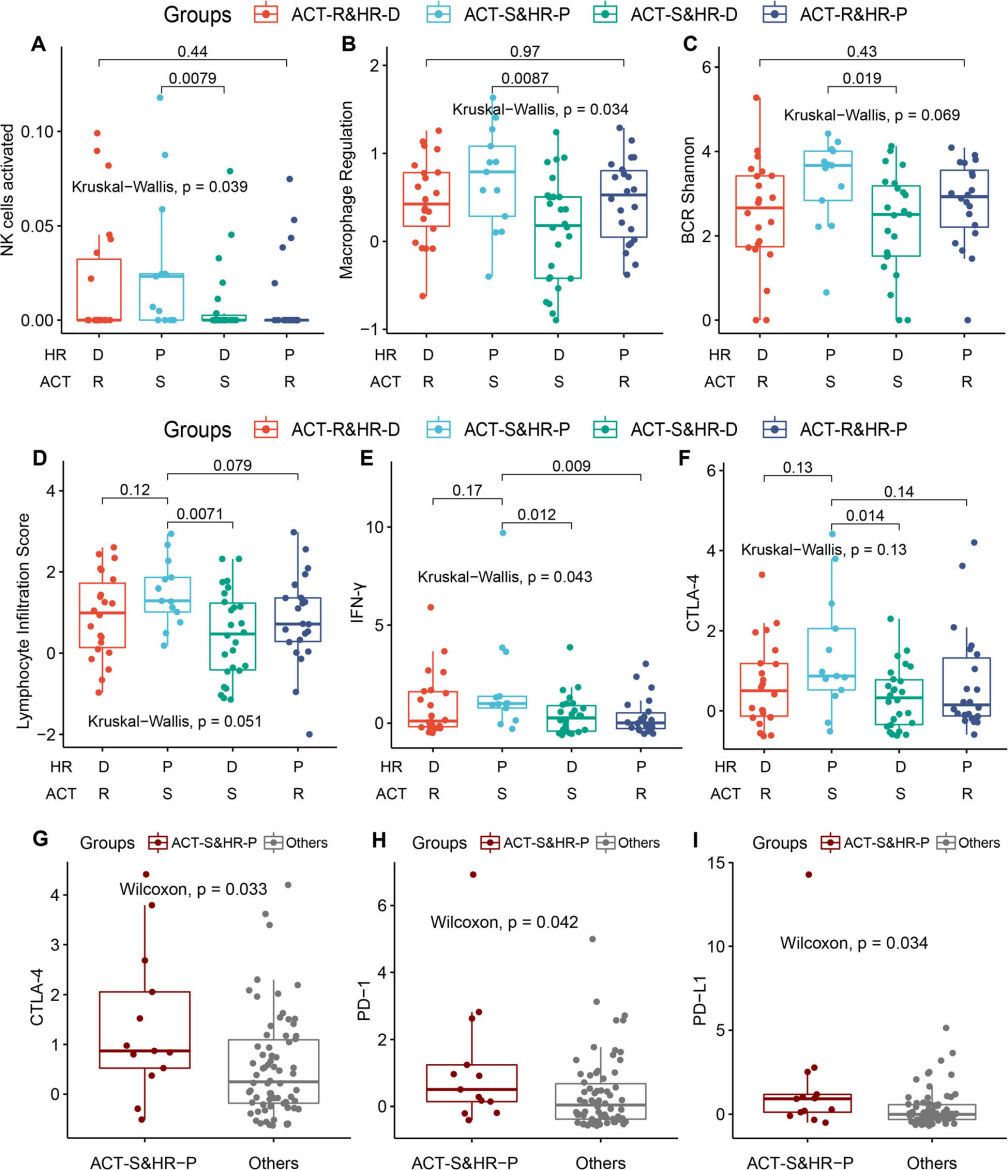
Analysis of the immune microenvironment mechanism in TNBC patients. **A**–**D** The box diagram showing the distribution of activated NK cells (**A**) and the immune molecular and cellular characteristics, including regulatory macrophages (Mregs, **B**), BCR repertoire diversity (**C**) and tumour-infiltrating lymphocytes score (**D**). **E**, **F** Under different HRD status and distinct ACT responses, the patients with IFN-γ activity (**E**) and the expression of *CTLA-4* (**F**) were diverse. *P*-value between the two (among multiple) groups was calculated by the Wilcoxon rank-sum (Kruskal-Wallis) test, the same below. ACT-S&HR-D: sensitive to ACT and HR deficiency (26 cases); ACT-S&HR-P: sensitive to ACT and HR proficiency (13 cases); ACT-R&HR-D: resistant to ACT and HR deficiency (22 cases); and ACT-R&HR-P: resistant to ACT and HR proficiency (22 cases). **G**–**I** The elevated expression of known cancer immunotherapy biomarkers, including *CTLA-4* (**G**), *PD-1* (**H**) and *PD-L1* (**I**), were correlated with ACT-sensitive and HR proficiency (ACT-S&HR-P). The others mean all samples except ACT-S&HR-P

Unrepaired DNA damage, especially HRD, modulates the tumour immune microenvironment through a range of molecular and cellular mechanisms [15, 16]. Low-dose doxorubicin and cyclophosphamide chemotherapy may stimulate anticancer immune responses and promote a more favourable tumour microenvironment [17, 18]. We speculated that the impact of HRD on the immune microenvironment was related to the ACT response in TNBC patients. As expected, we found that the effect of HRD on immune cell infiltration showed significant differences in distinct ACT response groups (Fig. 4, Additional file 1: Fig. S3). For example, in the ACT-sensitive (ACT-S) group, NK cells showed higher infiltration in HR-proficient (HR-P) samples (*P* = 0.0079, Wilcoxon rank-sum test, same below; Fig. 4A). In contrast, M0 macrophages and mast cells presented higher infiltration levels in HR-deficient (HR-D) samples (*P*-values were 0.02 and 0.012, respectively; Additional file 1: Fig. S3AB). Interestingly, differences in these immune cells were not observed in the ACT-resistant (ACT-R) group (Fig. 4A, Additional file 1: Fig. S3AB). Additionally, in the ACT-sensitive group, we found that regulatory macrophages (Mregs) were significantly activated in HR-proficient patients (*P* = 0.0087, Wilcoxon rank-sum test; Fig. 4B). These patients showed T cell/B cell receptor (TCR/BCR) repertoire diversity (*P*-values of 0.041 and 0.019, respectively; Fig. 4C, Additional file 1: Fig. S3C) and TCR/BCR richness (*P*-values of 0.036 and 0.018, respectively; Additional file 1: Fig. S3DE). Diversified TCR/BCR receptors are the basic attributes of an effective immune system, allowing T/B cells to target multiple types of endogenous or exogenous antigens [44, 45]. However, we did not find corresponding results in the ACT-resistant patient group (Fig. 4C, Additional file 1: Fig. S3CDE).

Studies have suggested that neoantigens presentation in tumours promotes the release of IFN-γ from tumour-infiltrating lymphocytes (TILs), and the released IFN-γ upregulates *PD-L1* expression in immune cells and tumours [46, 47]. We found that in the ACT-sensitive group, HR-proficient samples showed higher TIL infiltration (*P* = 0.0071, Wilcoxon rank-sum test, same below) and IFN-γ activity (*P* = 0.012) compared to HR-deficient samples (Fig. 4D, E). Among HR-proficient patients, those sensitive to ACT also showed higher TIL scores (*P* = 0.079, Wilcoxon rank-sum test, same below) and stronger IFN-γ activity (*P* = 0.009) than ACT-resistant patients (Fig. 4D, E). The patients with ACT-S&HR-P (sensitive to ACT and HR proficiency) were associated with higher IFN-γ activity (*P* = 0.011, Wilcoxon rank-sum test; Additional file 1: Fig. S4A) and lower TGF beta response (Additional file 1: Fig. S4B), implying an intense immune response in this subtype. Furthermore, for the known cancer immunotherapy biomarkers, we found that their expression levels were correlated with ACT response and HRD status (Fig. 4F, Additional file 1: Fig. S4CD). For example, significantly higher expression levels of *CTLA-4*, *PD-1* and *PD-L1* were found in ACT-S&HR-P patients (*P* < 0.05, Wilcoxon rank-sum test; Fig. 4G–I). In particular, patients with HR deficiency showed higher TMB (*P* = 0.0031, Wilcoxon rank-sum test; Additional file 1: Fig. S4E) and neoantigen levels (*P* = 0.0018, Wilcoxon rank-sum test; Additional file 1: Fig. S4F), which may be related to the fact that HRD exacerbates DNA DSBs, thereby promoting genome instability and causing the release of molecular antigens [48].

### Immune checkpoint blockade as an optional treatment for patients with ACT-S&HR-P subtype

**Fig. 5.**
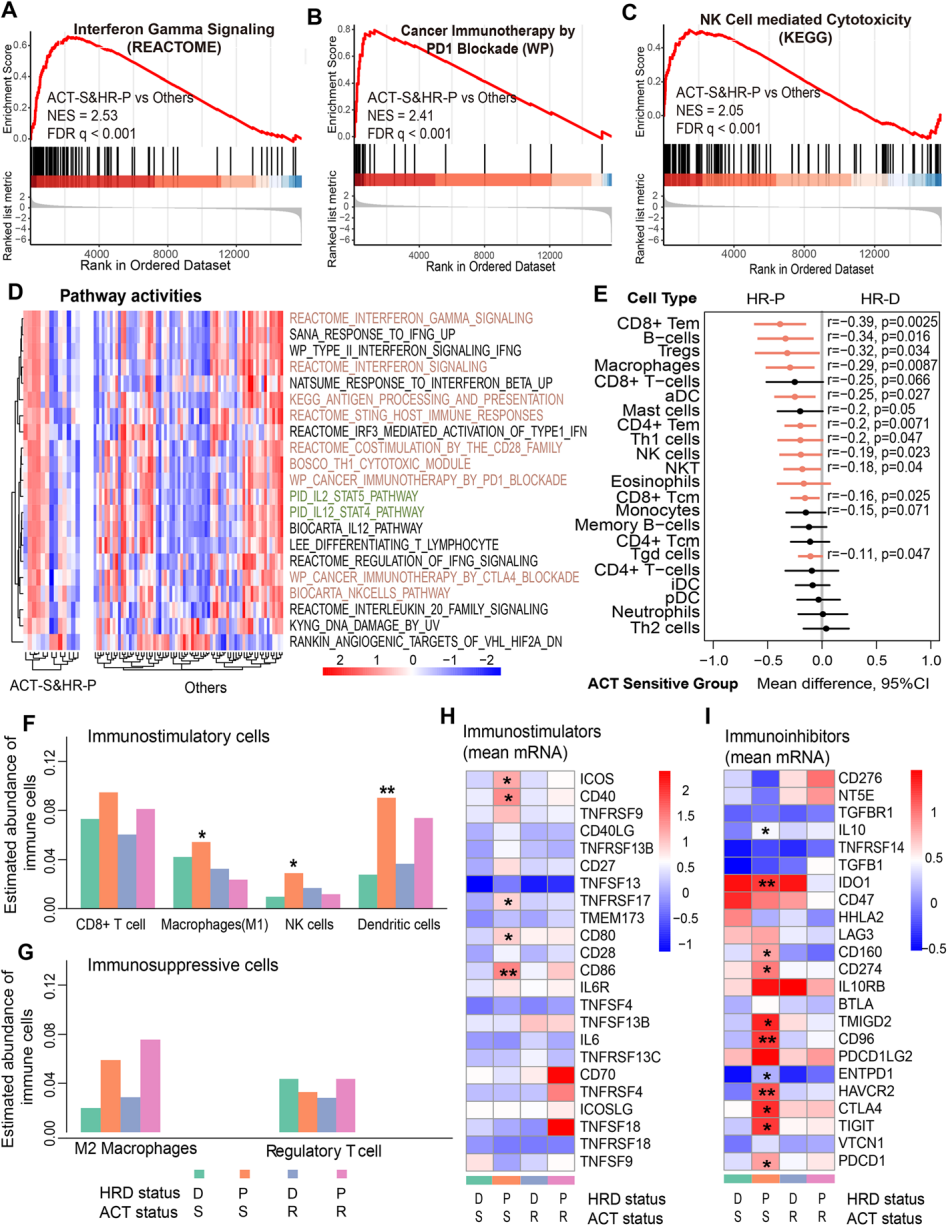
Potential application of immune checkpoint blockade in the ACT-S&HR-P subtype. **A**–**C** Representative gene set enrichment analysis plot showing upregulated interferon gamma signalling (**A**), cancer immunotherapy by *PD-L* blockade (**B**) and natural killer cell–mediated cytotoxicity (**C**) in the ACT-S&HR-P (sensitive to ACT and HR proficiency) subtype versus the other subtypes. NES, normalized enrichment score. **D** Pathway activity in ACT-S&HR-P patients and other patients. The highlighted pathways are indicated as being focused on in this research. **E** In the ACT-sensitive group, immune cell activity scores were higher in the HR-proficient samples. The dots depict the mean difference of immune cell activity scores in HR-deficient samples compared to HR-proficient samples, and the lines show the 95% confidence interval (CI) for the difference. *P* < 0.05 was considered significant (red colour), Wilcoxon rank-sum test. See Figure S6 for the full name of immune cell types. **F**, **G** Relative number of immune-stimulatory cells (**F**) and immune-suppressive cells (**G**) in the four TNBC subtypes calculated using the CIBERSORT algorithm. **H**, **I** The expression of immune stimulatory molecules (**H**) and immune checkpoint genes (**I**) in four TNBC subtypes. ***P < 0.001, **P < 0.01, *P < 0.05

The clinical and translational data indicated that short-term doxorubicin treatment may increase the likelihood of a response to *PD-1* blockade in TNBC [17]. Considering the results of our study, we postulated that ACT-S&HR-P patients may benefit from immune checkpoint blockade (ICB) therapy. To test this postulate, we performed GSEA on the C2 pathways from MSigDB (v7.2) (the “Methods” section). We found that the genes that were upregulated in ACT-S&HR-P patients were enriched in multiple immune response-related pathways, such as interferon-gamma signalling (NES = 2.53, FDR < 0.001; Fig. 5A, Additional file 1: Fig. S6A), interferon signalling (NES = 2.30, FDR < 0.001; Additional file 1: Fig. S5A) and type II interferon signalling IFN-γ (NES = 2.36, FDR < 0.001; Additional file 1: Fig. S5B). In addition, ICB-related pathways, including cancer immunotherapy by *PD-L* blockade (NES = 2.41, FDR < 0.001), the *CTLA-4* pathway (NES = 2.55, FDR < 0.001) and *CD28* family costimulation (NES = 2.45, FDR < 0.001), were related to the upregulated genes in this subtype (Fig. 5B, Additional file 1: Fig. S5CD). In particular, natural killer cell-mediated cytotoxicity (NES = 2.05, FDR < 0.001; Fig. 5C) and antigen processing and presentation (NES = 2.63, FDR < 0.001; Additional file 1: Fig. S5E) were also enriched to the upregulated genes in this subtype.

Consistent results were found using pathway activity. For example, cancer immunotherapy by *CTLA-4/PD-1* blockade was activated in ACT-S&HR-P patients (mean differences > 0, *P* < 0.05, Wilcoxon rank-sum test, the same below; Fig. 5D, Additional file 1: Fig. S6B). Similarly, the immune response-related pathways mentioned above also showed significant activation in this subtype, including interferon-gamma signalling (mean difference = 0.29, *P* = 0.0078), NK cell pathway (mean difference = 0.31, *P* = 0.011) and antigen processing and presentation (mean difference = 0.28, *P* = 0.0035) (Fig. 5D, Additional file 1: Fig. S6B). The *JAK-STAT* signalling pathway plays critical roles in the coordination of the immune system, especially for cytokine receptors, and it can regulate the polarization of helper T cells [14]. Our results showed that the genes upregulated in ACT-S&HR-P patients were enriched to the *JAK-STAT* signalling pathway (NES = 2.58, FDR < 0.001; Additional file 1: Fig. S5F). Additionally, the *IL2-STAT4* pathway (NES = 2.62, FDR < 0.001) and *IL2-STAT5* pathway (NES = 2.25, FDR < 0.001) showed correlations with the upregulated genes in this subtype (Additional file 1: Fig. S6A). Additionally, the pathways related to the inflammatory response, including the *IL2-STAT4/5* pathways presented higher activities in this subtype (Fig. 5D, Additional file 1: Fig. S6B). These results suggested that ACT-S&HR-P patients exhibit surprisingly elevated immune activities by activating the immune response pathways.

By analysing the types of immune cells [33], we found that among HR-proficient patients, both innate immune cells (such as activated DCs [aDCs], mast cells, macrophages and natural killer (T) cells [NKs/MKTs]) and adaptive immune cells (such as T helper 1 [Th1], CD8+ T central memory [Tcm], CD8+ T effector memory [Tem] and CD4+ Tem cells) were activated only in the ACT-sensitive group (*P* < 0.05, Wilcoxon rank-sum test, the same below; Fig. 5E, Additional file 1: Fig. S6CD). Similarly, the core biological pathways, including immune checkpoint (*P* = 0.027), CD8 T effector (*P* = 0.011) and antigen processing machinery pathways (*P* = 0.027), were also activated in ACT-S&HR-P patients (Additional file 1: Fig. S6EF). Additionally, analysing both immune cell infiltration and differential expression profiling revealed that the ACT-S&HR-P subtype was enriched for both immune-activated cells and immunostimulators. For instance, immunostimulatory cells, such as M1 macrophages, NK cells and dendritic cells, showed significantly higher activity in ACT-S&HR-P patients (Fig. 5F). However, the number of immune-suppressive cells was not elevated in this subtype (Fig. 5G). Expression profiling demonstrated that immunostimulators such as *CD40*, *CD86* and *ICOS* were significantly overexpressed in this subtype (Fig. 5H). The mechanisms by which ACT-S&HR-P patients show a stronger immune response likely involve the recruitment of immune-activated cells. In particular, our results revealed that immune inhibitors, especially *IDO1*, were significantly elevated in ACT-S&HR-P patients (Fig. 5I). This provides a valuable reference for additional reasonable immune checkpoint blockade therapeutics for TNBC patients with the ACT-S&HR-P subtype.

### Enhanced efficacy of identifying ACT responders by combining HRD and immune activation

**Fig. 6:**
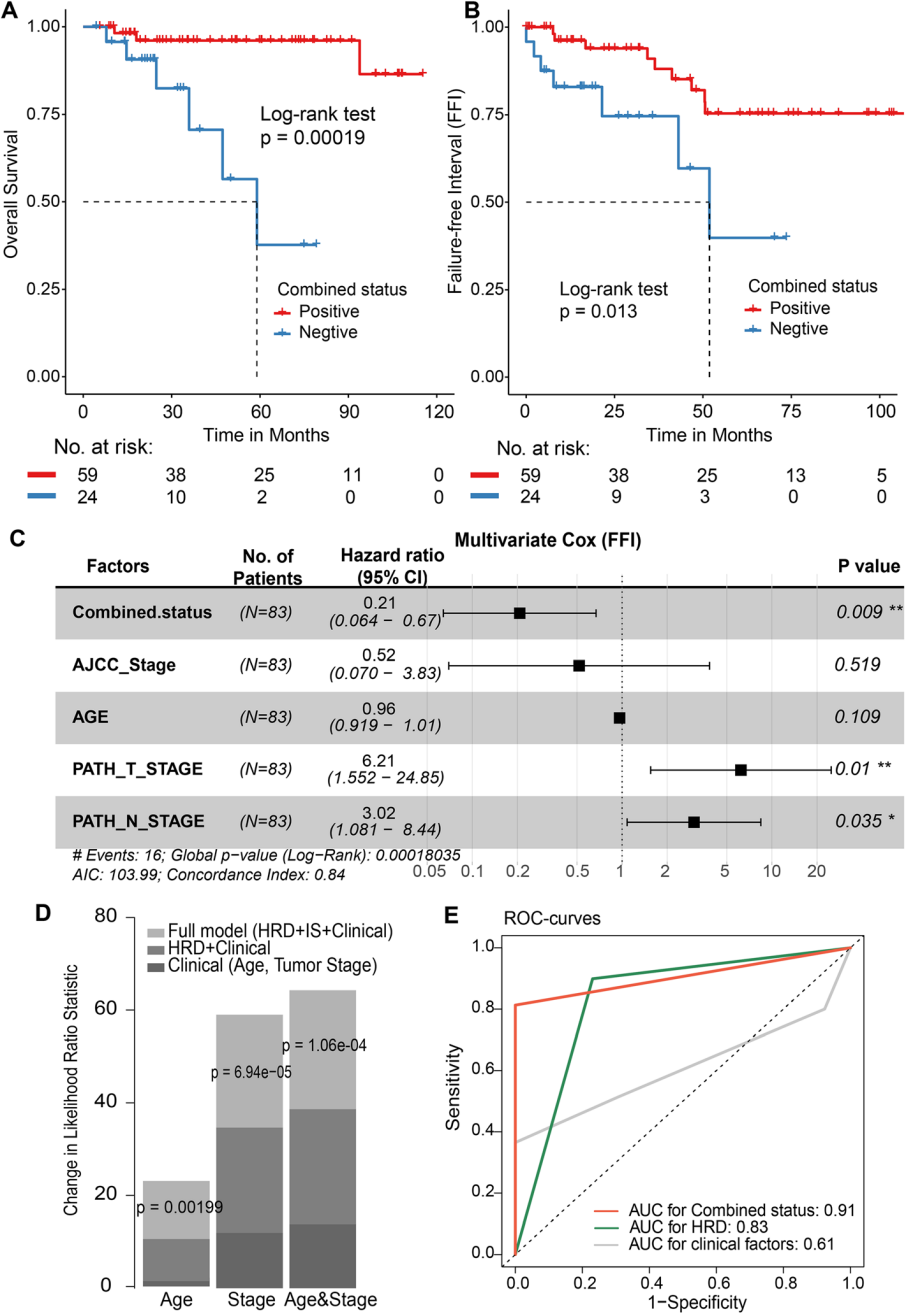
Combining HRD and immune activation enhances the efficacy of identifying ACT responders. **A**, **B** OS (**A**) and FFI (**B**) by the status of combined HRD and immune activation. Log-rank test. **C** Multivariate Cox analysis after correcting for clinical factors such as age, AJCC stage and TNM stage. The pathology M stage is not included in the model due to incomplete information. ***P < 0.001, **P < 0.01, *P < 0.05. **D** The estimated likelihood ratio (LR) statistic of a Cox model in clinical factors, such as age, stage, and age and stage was compared. The change of LR statistic as features were added to the model was assessed for significance. **E** The area under the curve (AUC) of the combined status (orange colour), HRD status alone (green colour) and only clinical factors (grey colour) in the patient’s 5-year survival time (FFI) were compared

The above findings implied that HRD and immune cell activity might synergistically affect the ACT response; thus, we wondered whether the combination of HRD and immune activation could improve the ACT chemotherapy response. We computed the immune score (IS) of patients based on the immune response pathways (the “Methods” section) and annotated the patients with the highest 25% IS as being positive for IS (IS+). Our study showed that combined positivity (i.e. positivity for HRD, IS, or both) was significantly associated with clinical benefit (*P* = 1.9e−04, log-rank test; Fig. 6A) with a hazard ratio of 0.037 (95% CI 0.0048–0.29, *P* = 0.002, Additional file 1: Fig. S7C) and prolonged DSS of patients (*P* = 0.018, Additional file 1: Fig. S7AB). More importantly, we found that the patients with combined positivity had a longer ACT failure-free interval (*P* = 0.013, log-rank test; Fig. 6B). After adjusting for clinical factors, the combined positivity was found to be a significantly independent prognostic factor (HR = 0.21, 95% CI 0.064–0.67, *P* = 0.009; Fig. 6C). The results were consistent with the known prognostic immune markers of breast cancer (Additional file 1: Table S3, Fig. S7D-F). Incorporating IS into Cox models fit with age, tumour stage, and age and tumour stage improved the predictive accuracy of FFI (*P* < 0.002, likelihood ratio test; Fig. 6D), which highlights the importance of the combination of HRD status and immune activities in ACT chemotherapy. Additionally, we found that the prognostic efficacy of combined status (AUC = 0.91) was better than that of HRD status alone (AUC = 0.83) or clinical factors alone (AUC = 0.61) (Fig. 6E). These results indicated the necessity of combining HRD status with tumour immunity, which improves the efficacy of identifying ACT responders in TNBC.

**Fig. 7:**
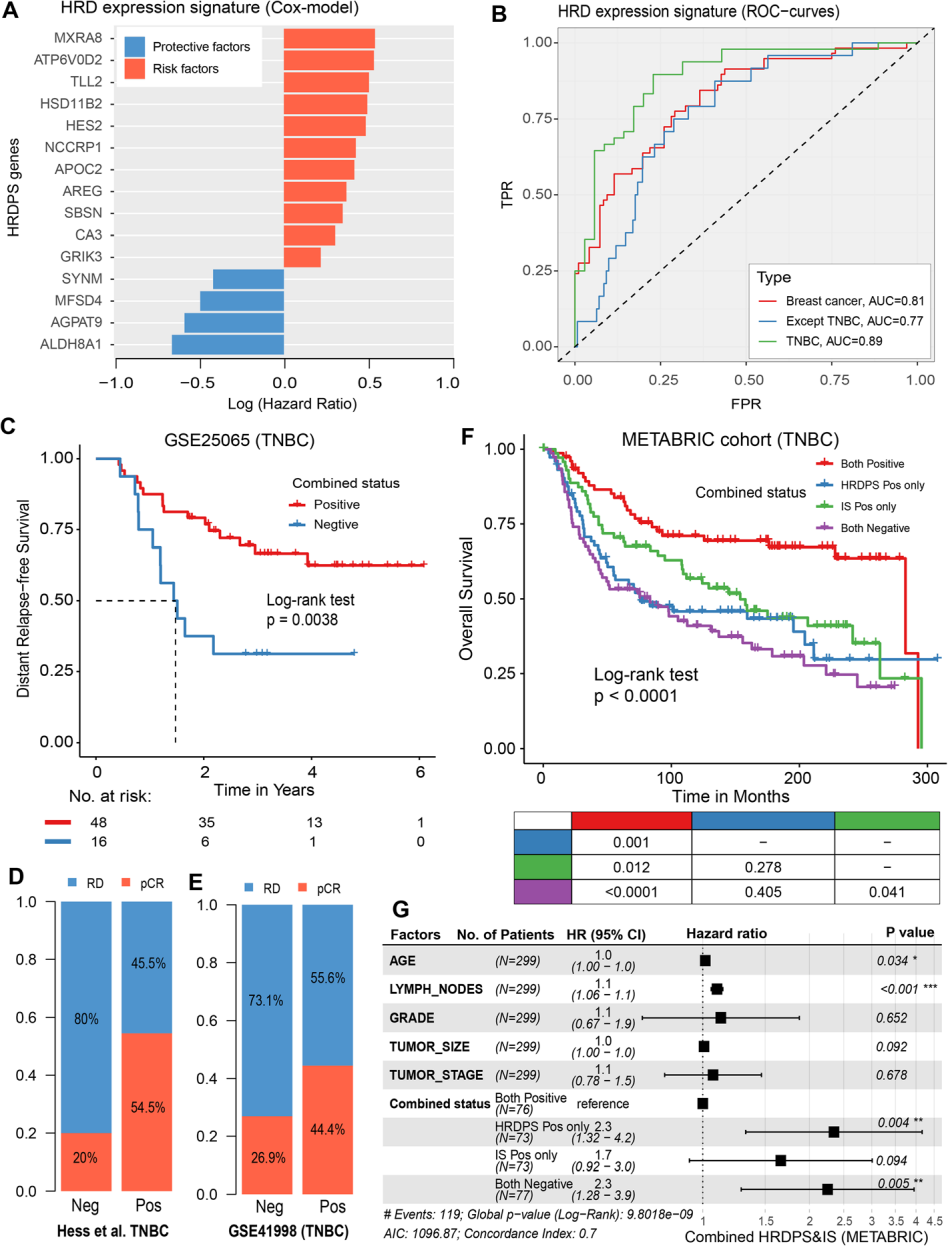
Combined status contributes to ACT response and clinical prognosis of TNBC patients. **A** Hazard ratio of HRD expression signature was calculated using Cox regression model. Log (hazard ratio) > 0 indicates a risk factor (red); log (hazard ratio) < 0 indicates a protective factor (blue). **B** Performance evaluation that expression signature predicts HRD using lasso logistic regression in diverse types of breast cancer samples, including TNBC samples (green), breast cancer samples (red) and breast cancer except for TNBC (blue). **C** Distant relapse-free survival (DRFS) by combined status in GSE25065 TNBC cohort. **D**, **E** The bar chart shows the proportion of pCR/RD samples with the combined status in Hess et al. TNBC cohort (**D**) and GSE41998 TNBC cohort (**E**). pCR, pathological complete response; RD, residual disease. Neg, negative; Pos, positive. **F** OS by combined status in the METABRIC cohort. The prognostic of HRDPS alone was shown in Additional file 1: Fig. S8C. **G** The forest plot shows the combined status as an independent prognostic factor for TNBC patients using multivariate Cox regression analysis (METABRIC cohort)

To further validate those findings in an independent dataset of TNBC cases, we developed an HRD expression signature that predicts ACT response (FFI) in TCGA TNBC cohorts (the “Methods” section). We identified 15 genes that were associated with FFI, including 4 that had a better ACT response and 11 that had a worse response in HR deficiency than in HR-proficient cases (Fig. 7A, Additional file 1: Table S4). The HRD expression signature showed excellent performance in reflecting the genomic HRD status applied to TNBC patients, as demonstrated by a receiver operating characteristic (ROC) curve with an AUC of 0.89 (Fig. 7B), which was superior to other types of breast cancer samples, including all BC patients (AUC = 0.81) and BC patients except TNBC (AUC = 0.77).

### The combined score contributes to the ACT response and clinical benefit of TNBC patients

We demonstrated that the combination of the HRD-related prognostic score (HRDPS) and IS was an effective way to predict TNBC patients who may achieve pCR to ACT chemotherapy, showing better prognosis in independent validation sets (Additional file 2: Table S5) [21, 22, 24]. Our results indicated that the patients with combined positivity had longer distant relapse-free survival (DRFS; *P* = 3.8e−3, log-rank test; Fig. 7C). After adjusting for clinical factors, combined negativity was a significant independent risk prognostic factor in TNBC patients with a hazard ratio of 6.4 (95% CI 2.38–17.1, *P* < 0.001) compared to combined positivity (Additional file 1: Fig. S8A). However, there was no statistical significance when using HRDPS alone, although patients with HRD-positive had better DRFS (*P* = 0.062, log-rank test; Additional file 1: Fig. S8B). In addition, the patients with combined positivity had higher pCR rates of ACT in two independent validation sets (54.5% for Hess et al. TNBC, 44.4% for GSE1998 TNBC) compared with combined negativity cases (20% for Hess et al. TNBC, 26.9% for GSE1998 TNBC; Fig. 7D, E).

Furthermore, we analysed the impact of HRDPS, IS and combined status on the prognosis of TNBC patients who were treated with chemotherapy [23]. The results showed that the combination of HRD status and tumour immune activation showed a strong correlation with patient OS (*P* < 0.0001, log-rank test; Fig. 7F). The patients with positivity for both factors showed the longest OS compared with those with other statuses (*P* < 0.0001 compared with both negative, *P* = 0.001 compared with HRDPS-positive only and *P* = 0.012 compared with IS-positive only, log-rank test; Fig. 7F). Multivariate Cox regression showed that the patients who were negative for both (HR = 2.3 95% CI 1.26–3.9, *P* = 0.005) and HRDPS-positive only (HR = 2.3 95% CI 1.32–4.2, *P* = 0.004) showed a significantly worse prognosis compared with patients who were positive for both (Fig. 7G).

Similarly, we acquired consistent results in two additional validation sets of TNBC patients who received ACT intervention [22, 25]. For example, the patients with positivity for both HRDPS and IS showed the longest DRFS (GSE25055; *P* = 0.007 compared with IS-positive only; *P* = 0.041 compared with HRDPS-positive only; *P* = 0.053 compared with both negative, log-rank test; Additional file 1: Fig. S9A) and the best survival outcomes for DSS (Chin et al.; *P* = 0.037 compared with HRDPS-positive only; *P* = 0.049 compared with both negative, log-rank test; Additional file 1: Fig. S9B). These results demonstrated that the combination of HRD and tumour immune activation can indeed contribute to the ACT chemotherapy response and clinical outcomes of TNBC patients.

## Discussion

In this study, we developed an integrated strategy to predict the ACT chemotherapy response through the combination of HR deficiency and immune activation in TNBC patients. Our method integrated multiomics data to ensure that we can not only characterize the HRD phenotype of TNBC using different methods but also analyse the effect on the ACT chemotherapy response in combination with the immune microenvironment. The presence of *BRCA1/2* mutations or tumour genomic instability (HRD score ≥ 42) is surrogate markers of HR deficiency [8, 10], and a positive immune score is a surrogate of interferon-primed immune checkpoints in the tumour microenvironment [49]. The presence of one or both tumour features was associated with longer FFI (HR = 0.21, *P* = 0.009) and OS (HR = 0.037, *P* = 0.002) in TNBC patients. A transcriptional HRD signature related to the ACT response was independently validated to be significantly associated with improved survival in the GEO cohort (*P* = 0.0038) and the METABRIC dataset (*P* < 0.0001) and increased ACT pCR rates of TNBC patients. These results are clinically relevant and suggest that combining HRD status and tumour immunity may aid in the selection of TNBC patients who would benefit from ACT chemotherapy.

TNBC is a breast cancer subtype with fairly abundant defects in DNA damage repair, especially HRD (approximately 67%) [8, 10], which encourages us to start from the perspective of DNA DSBs and develop treatment strategies that are sensitive to DNA damage inducers or DNA synthesis inhibitors [9, 10]. Mutations in *BRCA1/2* genes, especially germline variations, along with other Fanconi anaemia (FA) pathway genes (such as *NBN*, *RAD54L*, *ATM*), are prototypic molecular alterations that confer HRD in breast cancer [8]. Unfortunately, we cannot acquire information about germline *BRCA1/2* variation from public data resources. The HRD score is an algorithmic assessment of three measures of tumour genomic instability, namely, loss of heterozygosity, telomeric allelic imbalance and large-scale state transitions, which is a recognized indicator to characterize HRDs [10, 40]. *BRCA1* promoter hypermethylation, as an inactivator of epigenetic modifications, was associated with a gene expression profile similar to that of inherited *BRCA1* mutation-associated breast cancer [9, 41]. Mutational signature 3 is a genomic feature associated with failure of DNA DSB repair in breast cancer and is highly related to *BRCA1/2* variation [41]. We identified HR-deficient patients with *BRCA1/2* mutations or HRD scores ≥ 42 and indeed found that HR deficiency was associated with higher SBS3 activity, HRR gene mutations and *BRCA1* promoter hypermethylation.

**Fig. 8:**
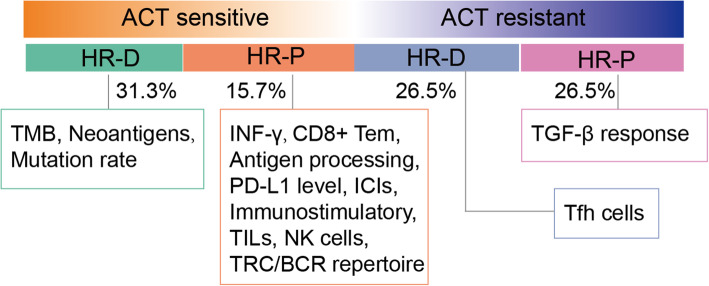
The characteristics summary of TNBC patients with HRD and ACT response. TMB, tumour mutation burden; ICIs, immune checkpoint inhibitors; TILs, tumour-infiltrating lymphocytes; NK cells, natural killer cells; TCR/BCR, T cell/B cell receptor; Tfh cells, T follicular helper cells

In agreement with previous observations, we found that HR deficiency was associated with better survival of TNBCs [8]. In addition, we further determined the FFI for TNBC patients who received ACT chemotherapy based on the period from the end of treatment to tumour progression/recurrence or death and found that HR deficiency was related to the patient durable response to ACT chemotherapy (*P* = 0.046). In cases of incomplete therapy response information from public databases, we explicitly classified TNBC patients as ACT-sensitive or ACT-resistant according to FFI and whether the tumour had progressed or complete responses after ACT treatment. This allowed us to further characterize the correlation between HRD status and ACT response. Considering the results of previous studies, the identification of TNBC patients who are sensitive to ACT is limited if only HRD status was considered [8]. The combined analysis of HRD status and the immune microenvironment provided us with an additional reference (Fig. 8), which made up for the limitations. Through transcriptional HRD signature analysis, we indicated that the HRD gene expression score was better at prognosis when combined with the immune score than their genetically determined HRD status. Our results demonstrated that the immunostimulatory cells and immune inhibitors (such as *PD-L1*, *CTLA-4* and *IDO1*) were significantly elevated in ACT-S&HR-P patients, providing additional rationale for the use of immune checkpoint blockade as a therapeutic approach.

The relative proportions of infiltrating immune cells were inferred based on gene expression profiles. However, mRNA only–based assessment of the immune infiltrate meets with several limitations. The architectural pattern and spatial distribution of TILs might be not captured by tumour transcriptomes. In addition, the differences of cellular RNA content may lead to estimation biases. And the calculation method particularly depends on the robustness of TIL marker genes [50, 51]. Therefore, it is necessary to consider the combination of immunohistochemistry to assess the immune infiltrate in future studies.

## Conclusions

In summary, our findings highlighted that HR deficiency could prolong ACT chemotherapy benefits (failure-free interval) and predicted an intensified ACT response in TNBC patients by combining HRD and immune activation. The combination synergistically contributes to the clinical outcomes of TNBC patients and enhances the efficacy of identifying ACT chemotherapy responders, which resolves the issue that ACT chemotherapy-sensitive patients cannot be clearly identified using HRD status alone. The combined status of HRD and immune activation based on gene expression assay can be used as a potential prognostic marker for TNBC patients, which suggests that combining the two types of characteristics has important application value in guiding the use of ACT chemotherapy in TNBCs. This provides a molecular basis for accurately identifying ACT chemotherapy responders and has prospective significance in clinical trials.

## Supplementary Information

**Additional file 1: Figure S1.** The mutations of homologous recombination repair genes in patients. **Figure S2.** Homologous recombination repair defects correlate with clinical benefits. **Figure S3.** Immune infiltration level of TNBC patients. **Figure S4.** Analysis of the immune microenvironment mechanism of TNBC patients. **Figure S5.** Representative gene set enrichment analysis plot. **Figure S6.** Immune checkpoints activated in the ACT-S&HR-P subtype. **Figure S7.** Combining HRD and immune checkpoints correlates with clinical benefits. **Figure S8.** HRD status and prognosis of TNBC patients. **Figure S9.** Combined status contributes to prognosis of TNBC patients. **Table S1.** Basic data information of TNBC patients. **Table S2.** Immune activation-related pathways and their genes. **Table S3**. Immune markers related to breast cancer. **Table S4**. Cox model results of HRD expression signature.

**Additional file 2: Table S5.** The values for HRD score (transcriptional HRD signature) and immune score of TNBC patients.

## Data Availability

All of the data we used in this study were publicly available as described in the “Methods” section.

## Abbreviations

ACT: Anthracycline, cyclophosphamide and taxane
AUC: Area under the curve
CIs: Confidence intervals
DEGs: Differentially expressed genes
DSBs: Double-strand breaks
DSS: Disease-specific survival
ER: Oestrogen receptors
FDR: False discovery rate
FFI: Failure-free interval
GEO: Gene Expression Omnibus
GSEA: Gene set enrichment analysis
HER2: Human epidermal growth factor receptor 2
HRD: Homologous recombination repair deficiency
HRDPS: HRD-related prognostic score
ICB: Immune checkpoint blockade
ICIs: Immune checkpoint inhibitors
IHC: Immunohistochemistry
IS: Immune score
LOH: Loss of heterozygosity
LST: Large-scale transitions
METABRIC: Molecular Taxonomy of Breast Cancer International Consortium
ntAI: Telomeric allelic imbalances
OS: Overall survival
pCR: Pathological complete response
PR: Progesterone receptors
PS: Prognostic score
RD: Residual disease
ROC: Receiver operating characteristic
TCGA: The Cancer Genome Atlas
TLI: Tumour lymphocyte infiltration
TMB: Tumour mutation burden
TNBC: Triple-negative breast cancer
TSS: Transcription start site
WES: Whole-exome sequencing
