# Supplementary material for: Combined homologous recombination repair deficiency and immune activation analysis for predicting intensified responses of anthracycline, cyclophosphamide and taxane chemotherapy in triple-negative breast cancer
Source: BMC Med. 2021 Sep 1;19:190. doi: 10.1186/s12916-021-02068-4 (PMC8408988; doi:10.1186/s12916-021-02068-4)
Supplement: Supplementary file 1 — Additional file 1: Figure S1. The mutations of homologous recombination repair genes in patients. Figure S2. Homologous recombination repair defects correlate with clinical benefits. Figure S3. Immune infiltration level of TNBC patients. Figure S4. Analysis of the immune microenvironment mechanism of TNBC patients. Figure S5. Representative gene set enrichment analysis plot. Figure S6. Immune checkpoints activated in the ACT-S&HR-P subtype. Figure S7. Combining HRD and immune checkpoints correlates with clinical benefits. Figure S8. HRD status and prognosis of TNBC patients. Figure S9. Combined status contributes to prognosis of TNBC patients. Table S1. Basic data information of TNBC patients. Table S2. Immune activation-related pathways and their genes. Table S3. Immune markers related to breast cancer. Table S4. Cox model results of HRD expression signature. [file 12916_2021_2068_MOESM1_ESM.pdf]

# Combined homologous recombination repair deficiency and immune activation analysis for predicting intensified responses of anthracycline, cyclophosphamide and taxane chemotherapy in triple-negative breast cancer

Gaoming Liao[1,$], Zedong Jiang[1,$], Yiran Yang[1,$], Cong Zhang[2], Meiting Jiang[3], Jiali Zhu[1], Liwen Xu[1], Aimin Xie[1], Min Yan[1], Yunpeng Zhang[1,*], Yun Xiao[1,4,*], Xia Li[1,4,*]

[1]College of Bioinformatics Science and Technology, Harbin Medical University, Harbin, Heilongjiang 150081, China.

[2]Department of Ultrasonic Medicine, the First Affiliated Hospital of Harbin Medical University, Harbin, Heilongjiang 150010, China.

[3]Key Laboratory of University in Heilongjiang Province, Department of Pharmacy, the Second Affiliated Hospital of Harbin Medical University, Harbin 150086, China.

[4]Key Laboratory of Cardiovascular Medicine Research, Harbin Medical University, Ministry of Education, Harbin, Heilongjiang 150081, China.

*To whom correspondence should be addressed: lixia@hrbmu.edu.cn (Xia Li), xiaoyun@ems.hrbmu.edu.cn (Yun Xiao), zhangyp@hrbmu.edu.cn (Yunpeng Zhang).

[$]Gaoming Liao, Zedong Jiang, Yiran Yang contributed equally to this work as first author.

# Supplementary Figures

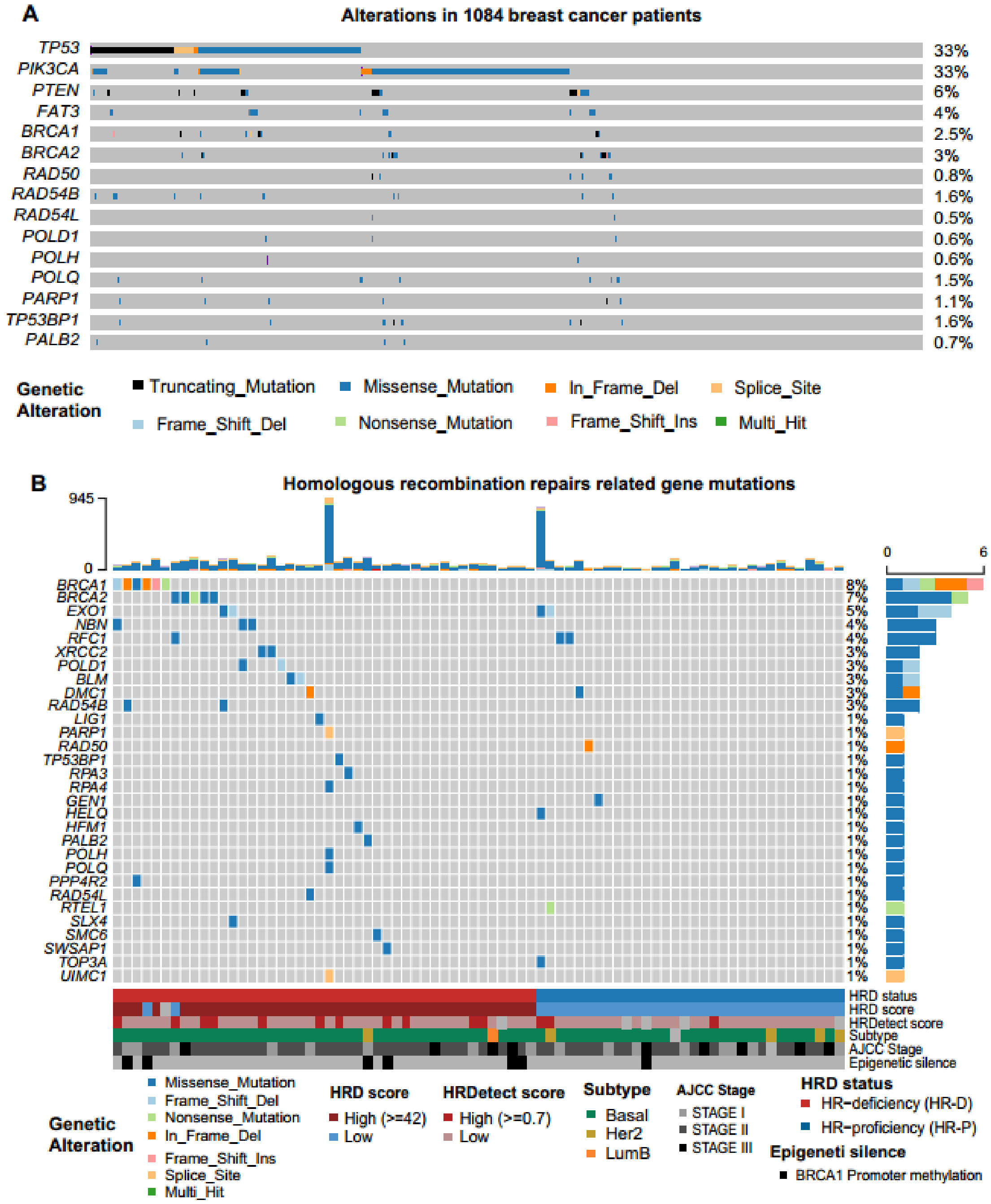


**Supplementary Figure S1. The mutations of homologous recombination repair genes in patients. A.** The mutations of the concerned genes in all breast cancers of the Cancer Genome Atlas (TCGA) (n = 1084) (1). **B**, The mutations of homologous recombination repair (HRR) genes in TNBC patients (n = 83). Positive HRD status (HR-deficiency) was defined as either a deleterious tumor *BRCA1/2* (tBRCA) mutation or a pre-defined HRD score >= 42(2, 3).

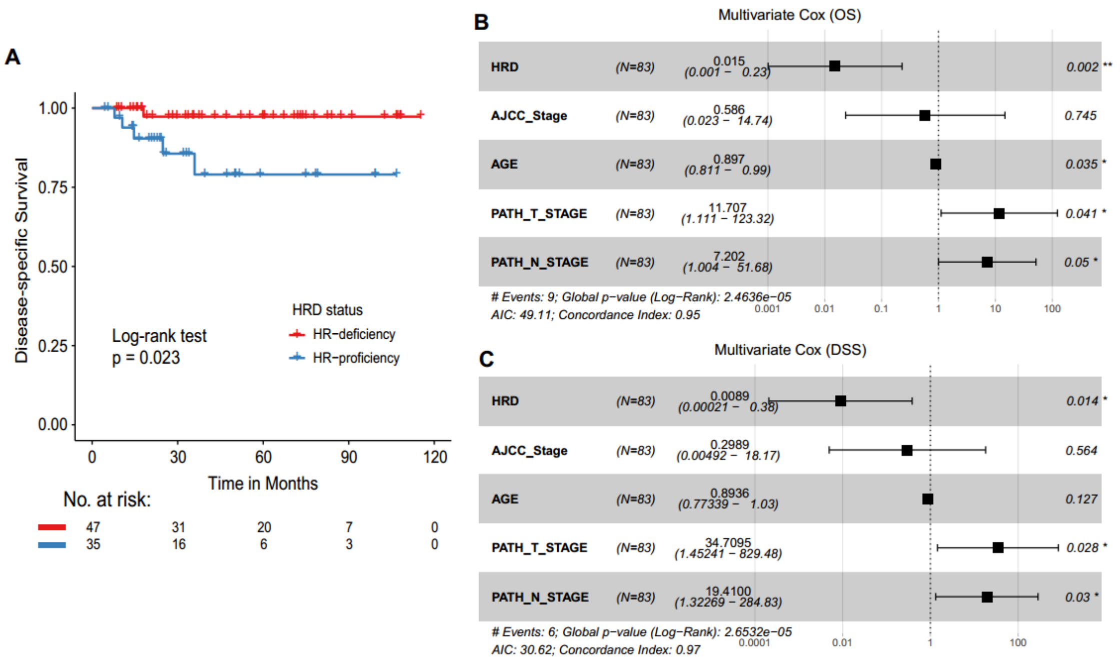


**Supplementary Figure S2. Homologous recombination repair defects correlate with clinical benefits. A**, Kaplan–Meier graphs of HRD status on disease-specific survival (DSS). **B-C**, Forest plot illustrating the HR (95% CI) for overall survival (OS, **B**) and DSS (**C**) calculated using the multivariate Cox proportional hazard models. HR, hazard ratios; CI, confidence interval.

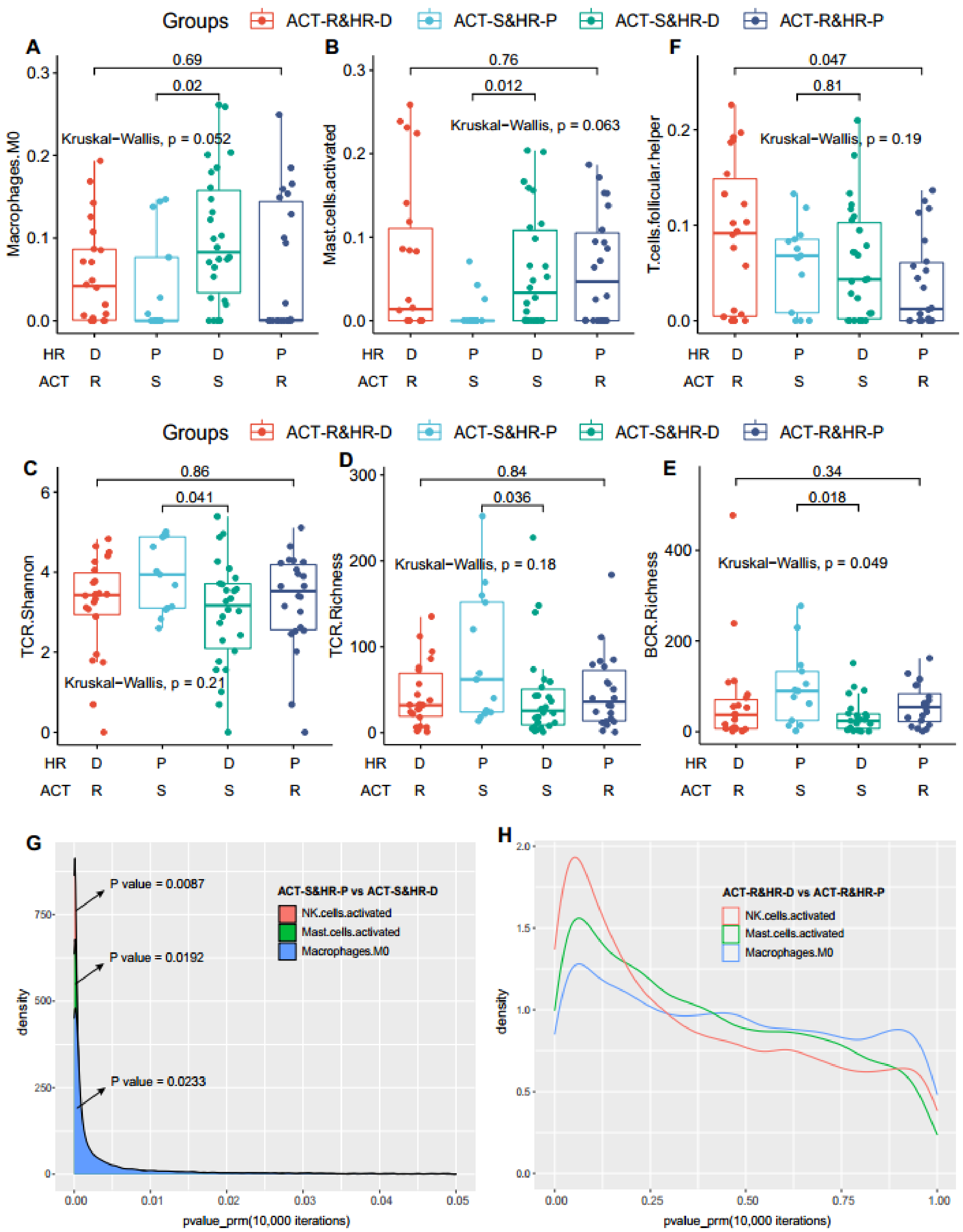


**Supplementary Figure S3. Immune infiltration level of TNBC patients. A-F**, The box diagram showing the distribution of immune molecular and cellular characteristics in the groups of different HRD statuses and distinct ACT responses, including activated macrophages M0 (**A**) and mast cells (**B**), TCR repertoire diversity (**C**), TCR richness (**D**), BCR richness (**E**) and follicular helper T (Tfh) cells (**F**). ACT-S&HR-D: sensitive to ACT and HR-deficiency, ACT-S&HR-P: sensitive to ACT and HR-proficiency, ACT-R&HR-D: resistant to ACT and HR-deficiency, ACT-R&HR-P: resistant to ACT and HR-proficiency. **GH**, For some comparisons (**G** for ACT-S&HR-P vs ACT-S&HR-D, and **H** for ACT-R&HR-D vs ACT-R&HR-P) in activated NK cells, M0 macrophages, and activated mast cells, a combinatorial method that

Wilcoxon's rank-sum test with continuity correction combined 10,000 iterations was be performed. The pvalue_prm: the p-value of comparison for each permutation. The P value (arrow) indicates the proportion of never meet the significance threshold (0.05) among 10,000 permutations.

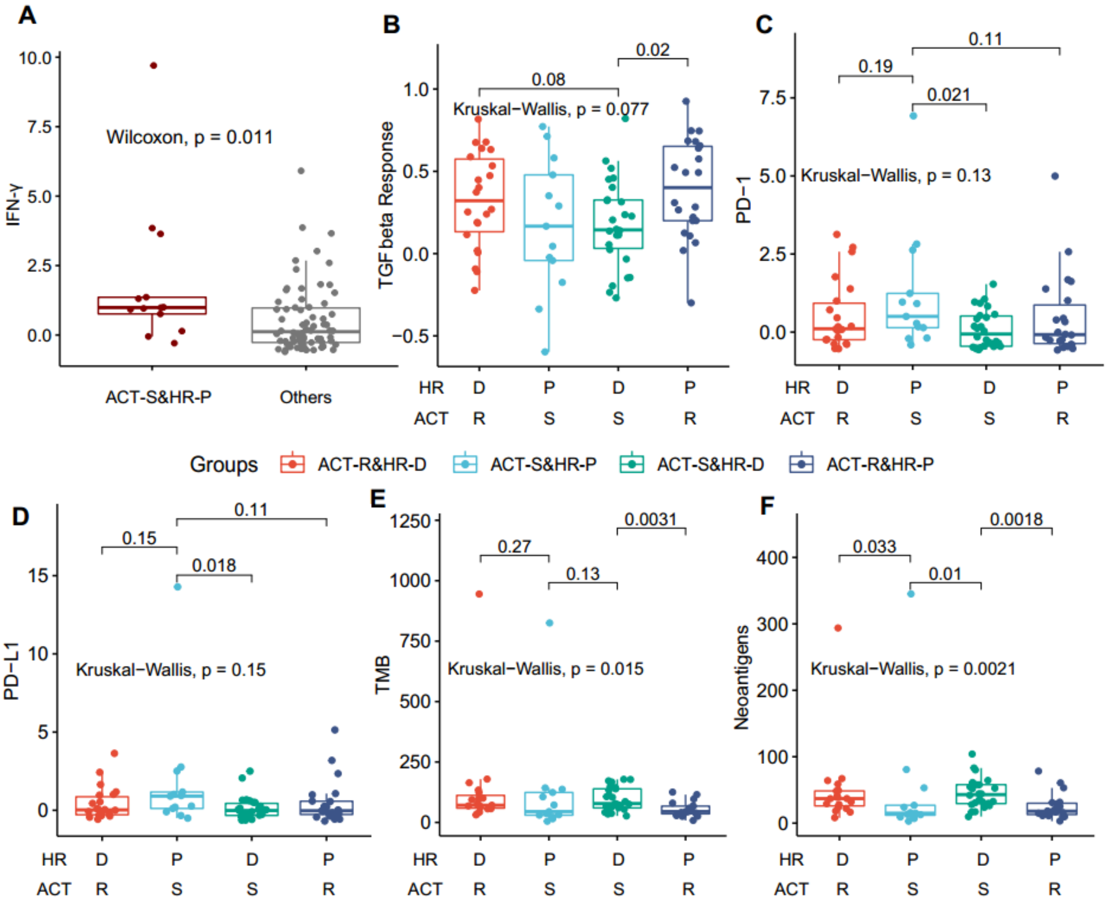


**Supplementary Figure S4. Analysis of the immune microenvironment mechanism of TNBC patients. A**, The elevated IFN-γ activity in patients with ACT-S&HR-P compared with other TNBCs. **B-F**, Under different HRD status and distinct ACT responses, the patients with TGF beta response (**B**), *PD-1* expression (**C**), *PD-L1* expression (**D**), tumor mutation burden (**E**) and neo-antigens (**F**) were diverse. Kruskal-Wallis test. ACT-S&HR-D: sensitive to ACT and HR-deficiency, ACT-S&HR-P: sensitive to ACT and HR-proficiency, ACT-R&HR-D: resistant to ACT and HR-deficiency, ACT-R&HR-P: resistant to ACT and HR-proficiency.

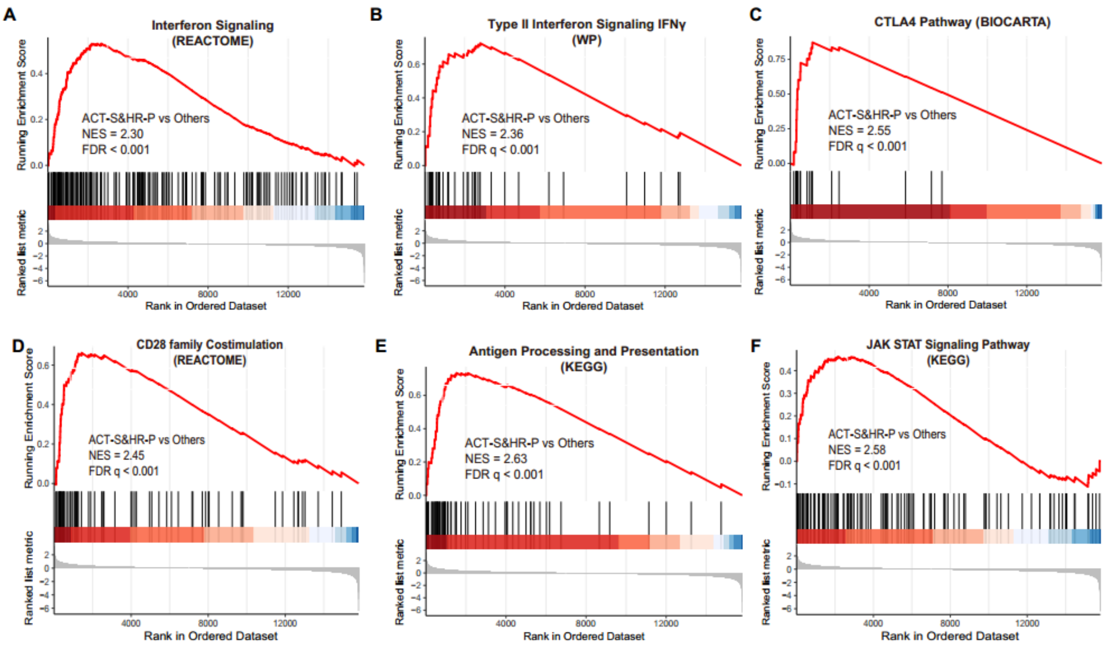


**Supplementary Figure S5. Representative gene set enrichment analysis plot.** These plots showing upregulated pathways in the ACT-S&HR-P (sensitive to ACT and HR-proficiency) subtype versus the other subtypes. The pathways are marked on the graph, including interferon signaling (**A**), type II Interferon signaling IFN-γ (**B**), *CTLA-4* pathway (**C**) and *CD28* family co-stimulation (**D**), antigen processing and presentation (**E**) and *JAK-STAT* signaling pathway (**F**).

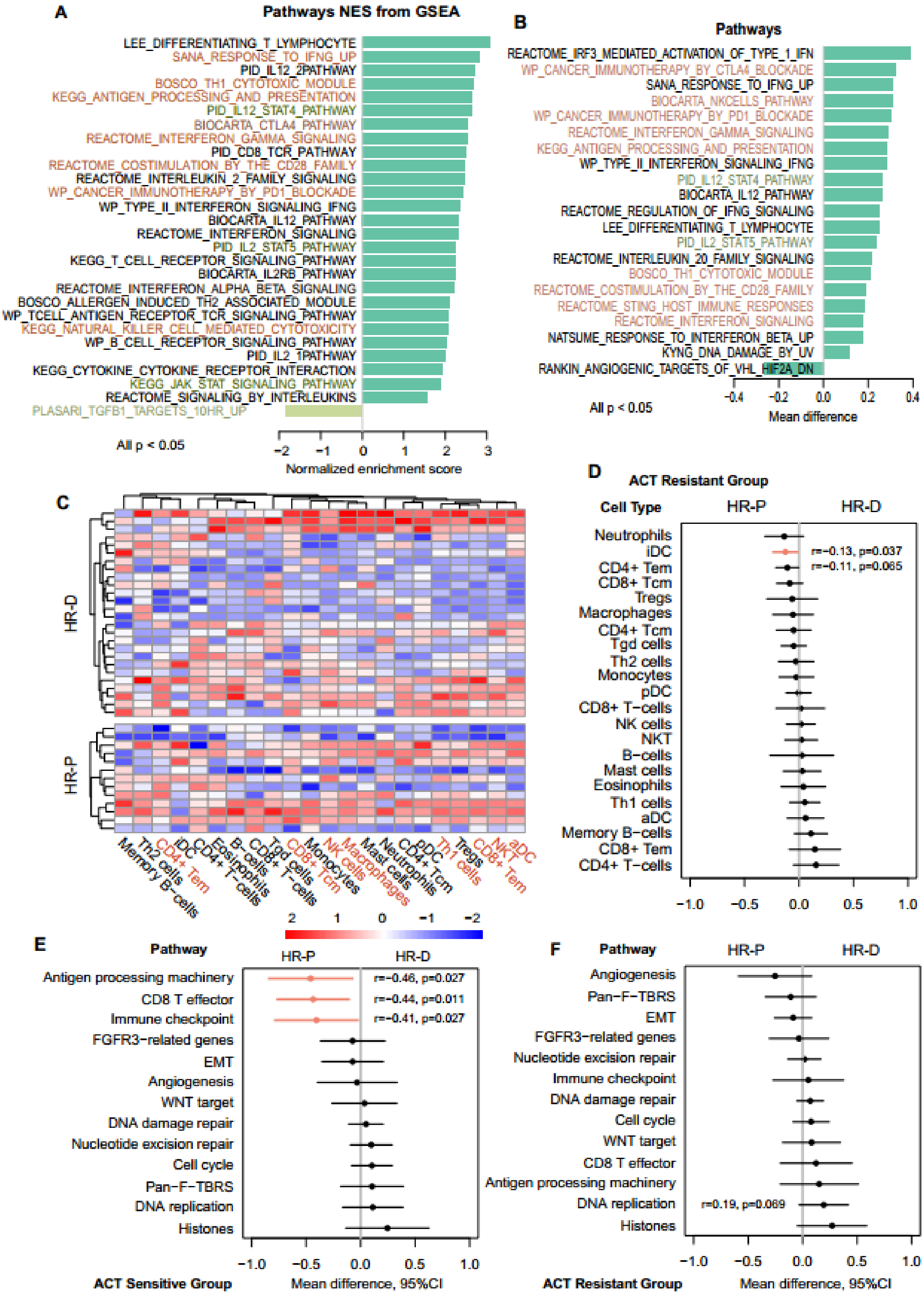


**Supplementary Figure S6. Immune checkpoints activated in the ACT-S&HR-P subtype. AB**, The histogram shows the NES (MSigDB v7.2, C2) using gene set enrichment analysis (**A**) and the mean difference of pathway activity in the ACT-S&HR-P (sensitive to ACT and HR-proficiency) subtype versus the other subtypes using GSVA (**B**). NES, Normalized enrichment score. **C**, In the ACT-sensitive group, the distribution of immune cell activity scores in patients with HR-deficiency (HR-D)

and HR-proficiency (HR-P). The highlighted cell types are indicated as being focused on in this research. **D**, In the ACT-resistant group, immune cell activity scores were no differences in the HR-D and HR-P samples, except immature dendritic cells (iDCs). aDCs, activated dendritic cells; Tem, Effector memory T cells; Tcm, Central memory T cells; Tregs, Regulatory T cells; Tgd cells, Gammadelta T cells; Th1 cells, T helper type 1 cells; Th2 cells, T helper type 2 cells; pDC, Plasmacytoid dendritic cell; NK cells, Natural killer cells; NKT, Natural killer T. **EF**, The core biological pathway activities of patients in ACT sensitive group (**E**) and ACT resistant group (**F**), respectively. EMT, Epithelial-mesenchymal transition; Pan-F-TBRS, Pan-fibroblast TGF-β response signature. The dots depict the mean difference of immune cell activity scores in HR-deficiency samples compared to HR-proficiency, and the lines show the 95% confidence interval (CI) for the difference. P-value < 0.05 was considered significant (red color), Wilcoxon rank-sum test.

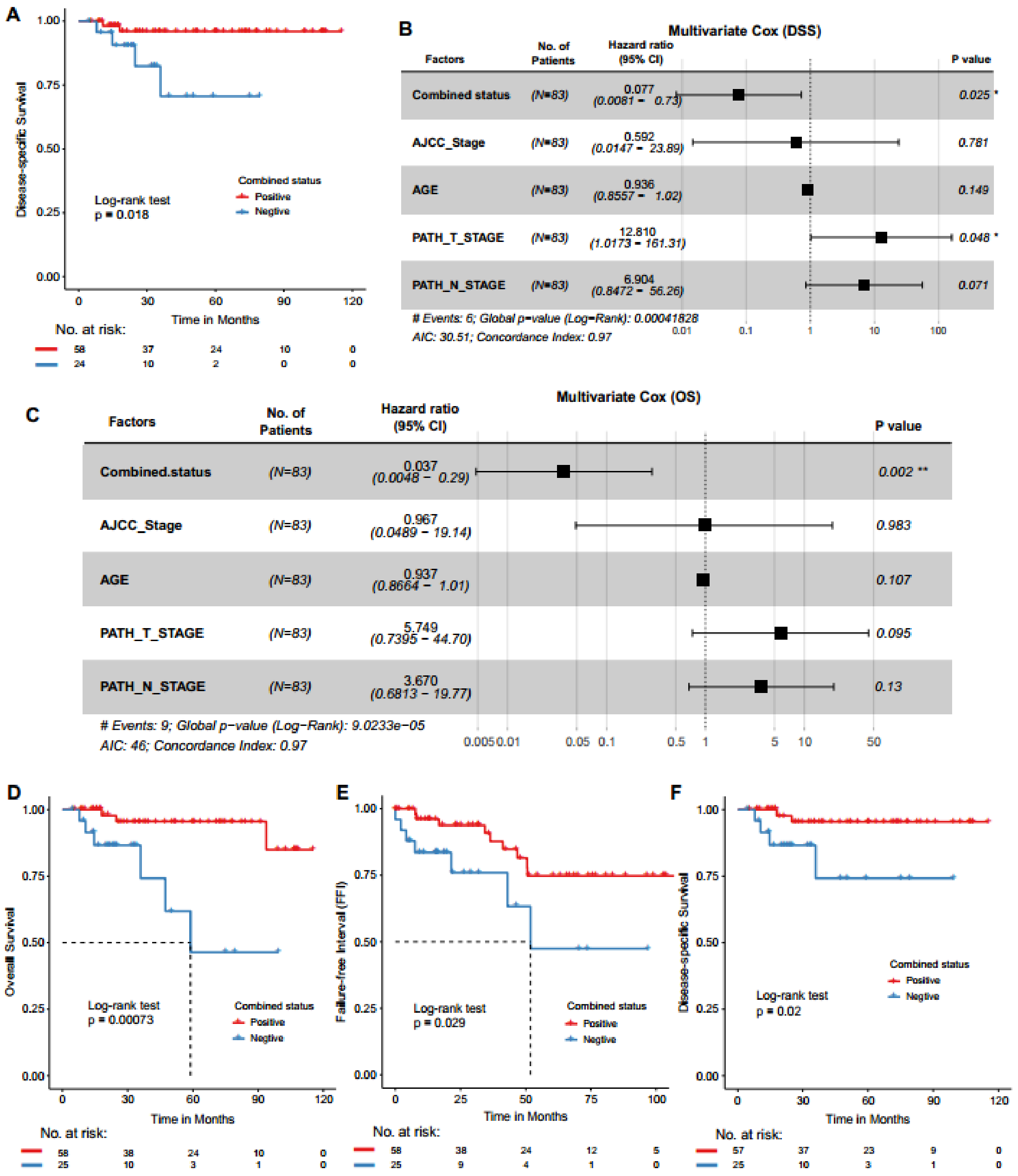

**Supplementary Figure S7. Combining HRD and immune checkpoints correlates with clinical benefits. A**, Kaplan–Meier graphs of combined HRD and immune checkpoints on disease-specific survival (DSS). Log rank test. **BC**, Forest plot illustrating the HR (95% CI) for DSS (**B**) and overall survival (OS, **C**) calculated using the multivariate Cox proportional hazard models. HR, hazard ratios; CI, confidence interval. **DEF**, Kaplan–Meier graphs of combined status on OS (**D**), failure-free interval (FFI, **E**), and DSS (**F**) using the prognostic immune markers of known breast cancer patients.

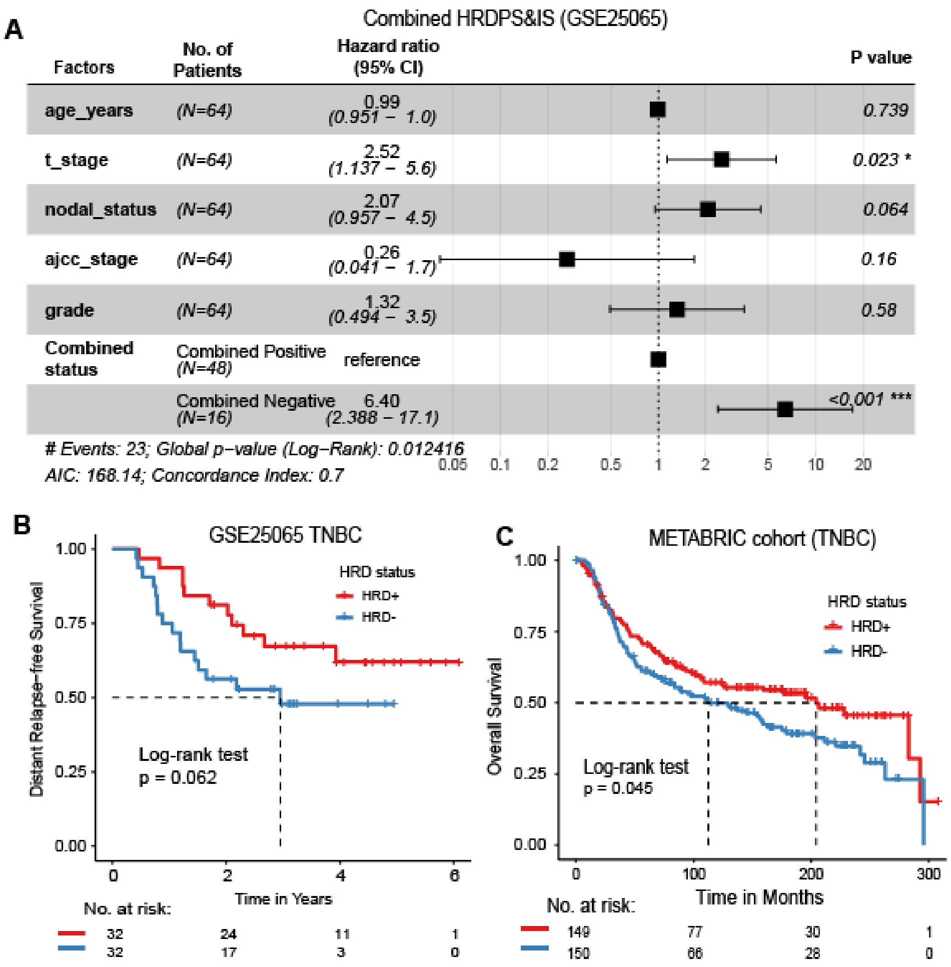


**Supplementary Figure S8. HRD status and prognosis of TNBC patients. A**, Forest plot illustrating the HR (95% CI) for distant relapse-free survival (DRFS) calculated using the multivariate Cox proportional hazard models, after correcting for clinical factors such as age, AJCC stage and nodal status, etc. HR, hazard ratios; CI, confidence interval. **BC**, DRFS and OS by the status of HRD status in GSE25065 (**B**) and METABRIC (**C**) TNBC cohort, respectively. Statistical significance was calculated using the log-rank test.

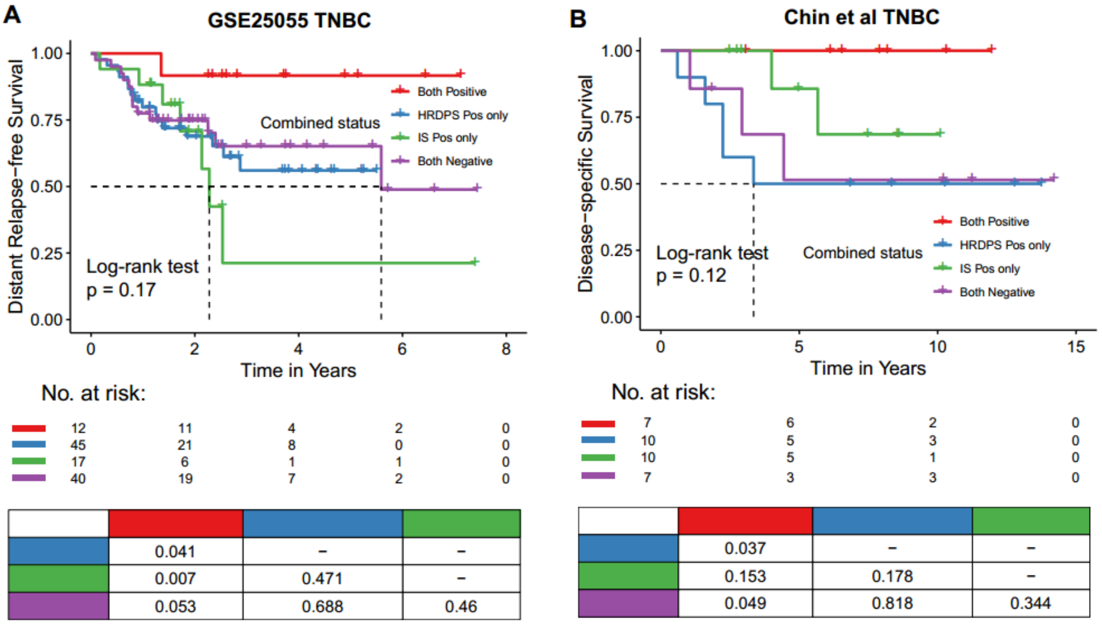


**Supplementary Figure S9. Combined status contributes to prognosis of TNBC patients. AB**, Kaplan–Meier graphs of distant relapse-free survival (DRFS) and disease-specific survival (DSS) by the status of combined HRD and immune checkpoints in GSE25055 (**A**) and Chin et al. (**B**) TNBC cohort, respectively. Statistical significance was calculated using the log-rank test.

# Supplementary tables

**Supplementary Table S1.** Basic data information of TNBC patients.

| Data resource (Dis /Val) | | No. of Samples | Survival / Response | Clinical phenotype |
|---|---|---|---|---|
| **TCGA** (Dis) | PanCancer Atlas Breast(1) | 83 | OS, DFS | Age, Stage, TNM stage |
| **GEO** (Val) | GSE25056(4) | 64 | DRFS | Age, Stage, Grade, Nodes status |
| | GSE25055(4) | 114 | DRFS | |
| | GSE41998(5) | 140 | Response | Age, Stage, Grade |
| **METABRIC** (Val)(6) | | 299 | OS | Age, Stage, Lymph Nodes, Tumor size |
| **UCSC Xena** (Val) | Chin2006(7) | 34 | DSS | Age, TNM stage, Grade |
| | Hess2006(8) | 27 | Response | Age, Grade, Race |

**TNBC**: Triple-negative breast cancer, **WES**: Whole Exome Sequencing, **OS**: Overall Survival, **DFS**: Disease Free Survival, **DRFS**: Distant Relapse-free Survival, **DSS**: Disease-specific Survival, **Dis**: Discovery datasets. **Val**: Validation datasets, **Response**: ACT chemotherapy status (pCR/RD).

**Supplementary Table S2.** Immune activation-related pathways and their genes.

| Pathways | Gene sets |
|---|---|
| **Immune checkpoint** | *CD274, CTLA4, HAVCR2, LAG3, PDCD1, PDCD1LG2, TIGIT* |
| **CD8 T effector** | *CD8A, CXCL10, CXCL9, GZMA, GZMB, IFNG, PRF1, TBX21* |
| ***CTLA4* Pathway** | *CD3G, CD3E, PIK3R1, ITK, CD3D, HLA-DRA, CD247, ICOS, CTLA4, LCK, CD86, HLA-DRB1, CD28, CD80* |
| **Cancer immunotherapy by *PD-1* blockade** | *IFNG, CD274, CD8A, BATF, CD3G, PDCD1, CD3E, CD3D, NFATC2, PDCD1LG2, LCK, CD8B, ZAP70, HLA-DRB1, HLA-A* |
| **NK cell mediated cytotoxicity** | *KLRC1, IFNG, FASLG, KLRC2, PIK3CG, PRF1, KLRD1, CD244, SH2D1B, KLRK1, SH2D1A, GZMB, PIK3R1, CD247, NFATC2, ITGAL, PRKCB, LCK, NCR3, CD48, FCGR3A, ZAP70, LCP2, FAS, PTK2B, HLA-B, SHC3, ITGB2, MICB, HLA-E, FCER1G, VAV1, HCST, HLA-A, TNFSF10, PTPN6, PIK3R5, HLA-G* |
| **Type II interferon signaling IFN- $\gamma$** | *IFNG, CXCL9, IRF1, GBP1, PSMB9, CYBB, STAT1, CIITA, IRF8, TAP1, JAK2, HLA-B, IRF2, IRF4, SOCS1, PRKCD, STAT2, IFNGR1, OAS1, PTPN11, CXCL10, IRF9, SPI1* |
| **Interferon gamma signaling** | *IFNG, GBP6, VCAM1, IRF1, GBP1, GBP5, HLA-DPA1, HLA-DQA2, GBP4, TRIM17, HLA-DRA, STAT1, HLA-DQB2, GBP2, HLA-DPB1, TRIM22, B2M, CIITA, PTAFR,* |

| | |
|---|---|
| | *IRF8, HLA-DQA1, FCGR1B, JAK2, FCGR1A, HLA-B, TRIM21, TRIM5, HLA-DRB1, TRIM34, HLA-F, HLA-E, CAMK2A, IRF2, IRF4, HLA-A, HLA-DQB1, SP100, PTPN6, HLA-G, SOCS1, TRIM2, HLA-H, IFI30, GBP3, TRIM8, PRKCD, HLA-C, HLA-DRB5, IRF3, IFNGR1, TRIM68* |
| **Response to IFN-γ up** | *IDO1, UBD, CD274, CXCL9, ATP6V0A4, APOL3, GBP1, HLA-DPA1, SAMD9L, GBP4, BATF2, STAT1, CD74, APOL1, CEACAM1, GIMAP7, TRIM22, SAMHD1, IL18BP, CX3CL1, HLA-DQA1, UBE2L6, NLRC5, HLA-DMA, C1S, PARP14, HLA-B, CXCL11, HLA-DRB1, CASP1, SSPN, LAP3, DTX3L, LGALS9, HLA-E, HLA-A, HLA-DQB1, PARP9, APOL4, TNFSF10, ETV7, VAMP5, DDX60, MX1, APOL2, GBP3, HLA-C, HLA-DRB5, RNF213, IFI35, OAS1, IFI44L, CXCL10, SERPING1, BST2* |
| **Regulation of IFN-γ signaling** | *IFNG, STAT1, JAK2, PTPN6, SOCS1, IFNGR1, PTPN11, IFNGR2, PIAS1* |

**Supplementary Table S3**. Immune markers related to breast cancer

| | |
|---|---|
| **Immune markers** (9) | *APOBEC3G, CCL5, CCR2, CD2, CD27, CD3D, CD52, CORO1A, CXCL9, GZMA, GZMK, HLA-DMA, IL2RG, LCK, PRKCB, PTPRC, SH2D1A* |

**Supplementary Table S4**. Cox model results of HRD expression signature.

| Prognostic factors | HR | 95% CI lower | 95% CI upper | P-value |
|---|---|---|---|---|
| *MXRA8* | 1.505 | 1.022 | 2.216 | 0.0383 |
| *ATP6V0D2* | 1.498 | 1.085 | 2.069 | 0.0141 |
| *TLL2* | 1.465 | 1.098 | 1.955 | 0.0095 |
| *HSD11B2* | 1.454 | 1.047 | 2.019 | 0.0253 |
| *HES2* | 1.444 | 1.152 | 1.81 | 0.0014 |
| *NCCRP1* | 1.381 | 1.113 | 1.712 | 0.0033 |
| *APOC2* | 1.372 | 1.033 | 1.823 | 0.0291 |
| *AREG* | 1.324 | 1.084 | 1.617 | 0.006 |
| *SBSN* | 1.302 | 1.099 | 1.543 | 0.0023 |
| *CA3* | 1.259 | 1.027 | 1.544 | 0.0265 |
| *GRIK3* | 1.18 | 1.001 | 1.391 | 0.0481 |
| *SYNM* | 0.728 | 0.584 | 0.909 | 0.005 |
| *MFSD4* | 0.687 | 0.483 | 0.978 | 0.0371 |
| *AGPAT9* | 0.64 | 0.419 | 0.978 | 0.0393 |
| *ALDH8A1* | 0.605 | 0.396 | 0.925 | 0.0202 |

# References


1. Hoadley KA, Yau C, Hinoue T, Wolf DM, Lazar AJ, Drill E, et al. Cell-of-Origin Patterns Dominate the Molecular Classification of 10,000 Tumors from 33 Types of Cancer. Cell. 2018;173:291-304 e6.
2. Sharma P, Barlow WE, Godwin AK, Pathak H, Isakova K, Williams D, et al. Impact of homologous recombination deficiency biomarkers on outcomes in patients with triple-negative breast cancer treated with adjuvant doxorubicin and cyclophosphamide (SWOG S9313). Annals of oncology : official journal of the European Society for Medical Oncology. 2018;29:654-60.
3. Loibl S, Weber KE, Timms KM, Elkin EP, Hahnen E, Fasching PA, et al. Survival analysis of carboplatin added to an anthracycline/taxane-based neoadjuvant chemotherapy and HRD score as predictor of response-final results from GeparSixto. Annals of oncology : official journal of the European Society for Medical Oncology. 2018;29:2341-7.
4. Hatzis C, Pusztai L, Valero V, Booser DJ, Esserman L, Lluch A, et al. A genomic predictor of response and survival following taxane-anthracycline chemotherapy for invasive breast cancer. Jama. 2011;305:1873-81.
5. Horak CE, Pusztai L, Xing G, Trifan OC, Saura C, Tseng LM, et al. Biomarker analysis of neoadjuvant doxorubicin/cyclophosphamide followed by ixabepilone or Paclitaxel in early-stage breast cancer. Clinical cancer research : an official journal of the American Association for Cancer Research. 2013;19:1587-95.
6. Curtis C, Shah SP, Chin SF, Turashvili G, Rueda OM, Dunning MJ, et al. The genomic and transcriptomic architecture of 2,000 breast tumours reveals novel subgroups. Nature. 2012;486:346-52.
7. Chin K, DeVries S, Fridlyand J, Spellman PT, Roydasgupta R, Kuo WL, et al. Genomic and transcriptional aberrations linked to breast cancer pathophysiologies. Cancer cell. 2006;10:529-41.
8. Hess KR, Anderson K, Symmans WF, Valero V, Ibrahim N, Mejia JA, et al. Pharmacogenomic predictor of sensitivity to preoperative chemotherapy with paclitaxel and fluorouracil, doxorubicin, and cyclophosphamide in breast cancer. Journal of clinical oncology : official journal of the American Society of Clinical Oncology. 2006;24:4236-44.
9. Yang B, Chou J, Tao Y, Wu D, Wu X, Li X, et al. An assessment of prognostic immunity markers in breast cancer. NPJ breast cancer. 2018;4:35.
